# Evaluation of genotype by environment interaction and adaptability in lowland irrigated rice hybrids for grain yield under high temperature

**DOI:** 10.1038/s41598-021-95264-4

**Published:** 2021-08-04

**Authors:** P. Senguttuvel, N. Sravanraju, V. Jaldhani, B. Divya, P. Beulah, P. Nagaraju, Y. Manasa, A. S. Hari Prasad, P. Brajendra, C. Gireesh, M. S. Anantha, K. Suneetha, R. M. Sundaram, M. Sheshu Madhav, M. D. Tuti, L. V. Subbarao, C. N. Neeraja, V. P. Bhadana, P. R. Rao, S. R. Voleti, D. Subrahmanyam

**Affiliations:** 1 Crop Improvement Section, ICAR - Indian Institute of Rice Research, Hyderabad, 500030 India; 2 ICAR - Indian Institute of Agricultural Biotechnology, Ranchi, 834010 India

**Keywords:** Heat, Plant breeding

## Abstract

Recent predictions on climate change indicate that high temperature episodes are expected to impact rice production and productivity worldwide. The present investigation was undertaken to assess the yield stability of 72 rice hybrids and their parental lines across three temperature regimes over two consecutive dry seasons using the additive main effect and multiplicative interaction (AMMI), genotype and genotype × environment interaction (GGE) stability model analysis. The combined ANOVA revealed that genotype × environment interaction (GEI) were significant due to the linear component for most of the traits studied. The AMMI and GGE biplot explained 57.2% and 69% of the observed genotypic variation for grain yield, respectively. Spikelet fertility was the most affected yield contributing trait and in contrast, plant height and tiller numbers were the least affected traits. In case of spikelet fertility, grain yield and other yield contributing traits, male parent contributed towards heat tolerance of the hybrids compared to the female parent. The parental lines G74 (IR58025B), G83 (IR40750R), G85 (C20R) and hybrids [G21 (IR58025A × KMR3); G3 (APMS6A × KMR3); G57 (IR68897A × KMR3) and G41 (IR79156A × RPHR1005)] were the most stable across the environments for grain yield. They can be considered as potential genotypes for cultivation under high temperature stress after evaluating under multi location trials.

## Introduction

Rice cultivation is adapted to a wide range of agro-climatic zones across the globe and staple diet for more than 3.5 billion people, provides 35–80% of total calorie uptake globally^1^. The global population is expected to be nine billion by 2050, which demands 60–110% more rice production than the present-day^2^. Recent estimates on climate change indicate that high-temperature episodes may affect the 20 Mha of the rice-growing area in Asian countries and, consequently, productivity by 14% in south Asia, 10% in East Asia and the Pacific, and 15% in sub-Saharan Africa. The Intergovernmental Panel on Climate Change (IPCC) projected severe crop damages by climate change, especially due to higher temperatures by 2 °C by 2050. The atmospheric CO_2_ concentration, which is likely to increase approximately 450 ppm by 2030 and 750 ppm by 2100 will result in earth mean surface temperature rise of 3.7–7.8 °C. Changing climate and genotype × environment interactions (GEI) affect the improvement of rice yield potential. Therefore, there is a prerequisite to adapt the available technologies and mitigate increasing temperature that could deliver estimated results.

The average annual yield increase has steadily declined from 3.2% per annum in 1960 to 1.5% in 2000 after the introduction of semi-dwarf rice varieties^3^. With available technologies, hybrid rice provides one important avenue for higher yields^4^. The advent of hybrid rice technology resulted in yield gain of 10–15% over high yielding pureline varieties^5^. Compared to conventional varieties, hybrids exhibit better vigour and yield potential^6^. The performance of the hybrids collectively dependent on the genotype, GEI of hybrids and identifying the best growing environments which helps in realizing the maximum grain yield.

Hybrids and their parental lines, must be evaluated across diverse environments to identify stable and high yield potential genotypes^7^. It was reported that over 80% of released hybrids in India are sensitive to heat and drought^8,9^. Heat tolerance in hybrid rice significantly correlates with their parents^10^. Male parent plays a major role in exhibiting tolerance level in resultant hybrids^11–13^. The heat stress index of F_1_ combinations was significantly correlated with the heat stress index of restorer lines but not with the heat stress index of maintainer lines^11^, whereas Gong^10^ reported that female parent influence the heat tolerance of three-line hybrid rice. The drought resistance of hybrids depends on selecting both the parental lines with better yield potential; high combining ability and drought tolerance contributing traits^14^. The indica hybrid rice combinations with pure indica male parent show higher heat tolerance than those with permeability japonica male parent^8^. Therefore, there is a need to develop parental lines (both maintainer and restorer lines) that are tolerant to heat and drought stresses. Parental lines and derivative hybrids, which perform stably at higher temperatures, are essential for development and wide-scale adoption of hybrid rice technology. In the case of hybrids developed for lowland irrigated cultivation, the genotype by season interaction and genotype by season by temperature interaction are critical to achieving potential grain yield. The selection of hybrids based on performance from a single environment is not considered effective, as grain yield of hybrids shows a complex quantitative inheritance and is heavily influenced by the environment. Therefore, it is imperative to execute the evaluation of rice hybrids for grain yield stability across multiple season and environments. Thus, the study was undertaken to evaluate rice hybrids for high yield and stability across seasons under variable temperature regimes.

## Results

### Yield and yield-related traits

In the present study, a total of 103 test entries were evaluated (through pooled analysis) and wide range of variation was recorded for all the traits namely, grain yield per plot (4506.86–9918.81 kg/ha), panicle weight (1.81–3.41 g), 1000 seed weight (15.33–21.11 g), single plant yield (14.4–30.87 g), spikelet fertility (75.04–90.61%), grains per panicle (107.78–225.71), panicle length (20.52–23.75 cm), number of productive tillers (9–13), plant height (77.66–112.77 cm) (Table 1). The replicated data from six environments were assessed and compared with the hybrid check (G98-KRH2) using significant pair-wise mean comparison.

**Table 1:** Phenotypic variability and Descriptive statistics of traits under study across the environments.

Variable	Min	Max	Mean	Median	Q1	Q3	Range	IQR
DFF (days)	78.89	116.94	107.58	108.21	103.81	111.83	38.05	8.02
PH (cm)	77.66	112.77	94.41	94.27	89.64	98.57	35.11	8.92
NRT	8.66	12.83	10.88	10.93	10.35	11.41	4.17	1.07
PL (cm)	20.52	23.75	22.29	22.31	21.95	22.66	3.23	0.72
PW (g)	1.81	3.41	2.75	2.75	2.58	2.92	1.6	0.34
GP	107.78	225.71	151.85	151.06	138.34	161.68	117.93	23.34
SF (%)	75.04	90.61	86.09	86.86	85.26	87.88	15.57	2.61
TW (g)	15.33	21.11	18.39	18.45	17.79	19.15	5.77	1.37
SPY (g)	14.4	30.87	19.91	19.69	17.46	21.58	16.47	4.12
YLD (kg)	4506.86	9918.81	6290.61	6245.19	5511.7	6847.26	5411.94	1335.56

Variable	Variance	SD	SEM	CV	CSS	UCSS	Skewness	Kurtosis
DFF (days)	31.77	5.64	0.56	5.24	3240.19	1,195,380.07	− 1.46	5.32
PH (cm)	53.68	7.33	0.72	7.76	5475.37	923,570.29	0.01	− 0.29
NRT	0.6	0.77	0.08	7.11	61.11	12,252.02	− 0.34	0.15
PL (cm)	0.38	0.61	0.06	2.75	38.28	51,227.19	− 0.23	0.19
PW (g)	0.08	0.28	0.03	10.08	7.82	784.67	− 0.34	0.92
GP	440.32	20.98	2.07	13.82	44,912.67	2,420,018.52	0.77	1.66
SF (%)	6.37	2.52	0.25	2.93	649.35	764,108.82	− 1.3	2.55
TW (g)	1.25	1.12	0.11	6.07	127.29	34,956.84	− 0.16	0.1
SPY (g)	8.73	2.95	0.29	14.84	890.29	41,733.6	0.71	0.88
YLD (kg)	877,525.8	936.76	92.3	14.89	89,507,631.4	4,165,404,837	0.7	1.14

Var, Variable; DFF, days to fifty percent flowering; PH, plant height; NRT, number of productive tillers; PL, panicle length; PW, panicle weight; GP, grains per panicle; SF, spikelet fertility; TW, 1000 seed weight; SPY, single plant yield; YLD, plot yield.

G65 (IR68897A × IR-66R) was found significantly better in terms of single plant yield and grain yield compared to hybrid check G99 (DRRH3), variety checks G96 (NDR359) and G100 (IR64), tolerant check G102 (Nagina22) and susceptible check G103 (Azucena). Among all the entries, G102 (Nagina22) recorded very early DFF and 30 days early than G99 (DRRH3) and G98 (KRH2) check entries. G12 (APMS6A × Akshayadhan), G13 (APMS6A × SG27-105), G32 (IR58025A × 363–5) and G71 (IR68897A × BK-49–180) were significantly shorter plant height in comparison with G103 (Azucena) and G96 (NDR359). G65 (IR68897A × IR-66R) and G60 (IR68897A × RPHR-517) recorded higher grain number per panicle as compared to checks G102 (Nagina22), G99 (DRRH3), G98 (KRH2) and G100 (IR64). G6 (APMS6A × RPHR-517) and G33 (IR58025A × RPHR611-1) have significantly higher spikelet fertility percentage than the tolerant check G102 (Nagina22) and hybrid check G99 (DRRH3).

**Figure 1:**
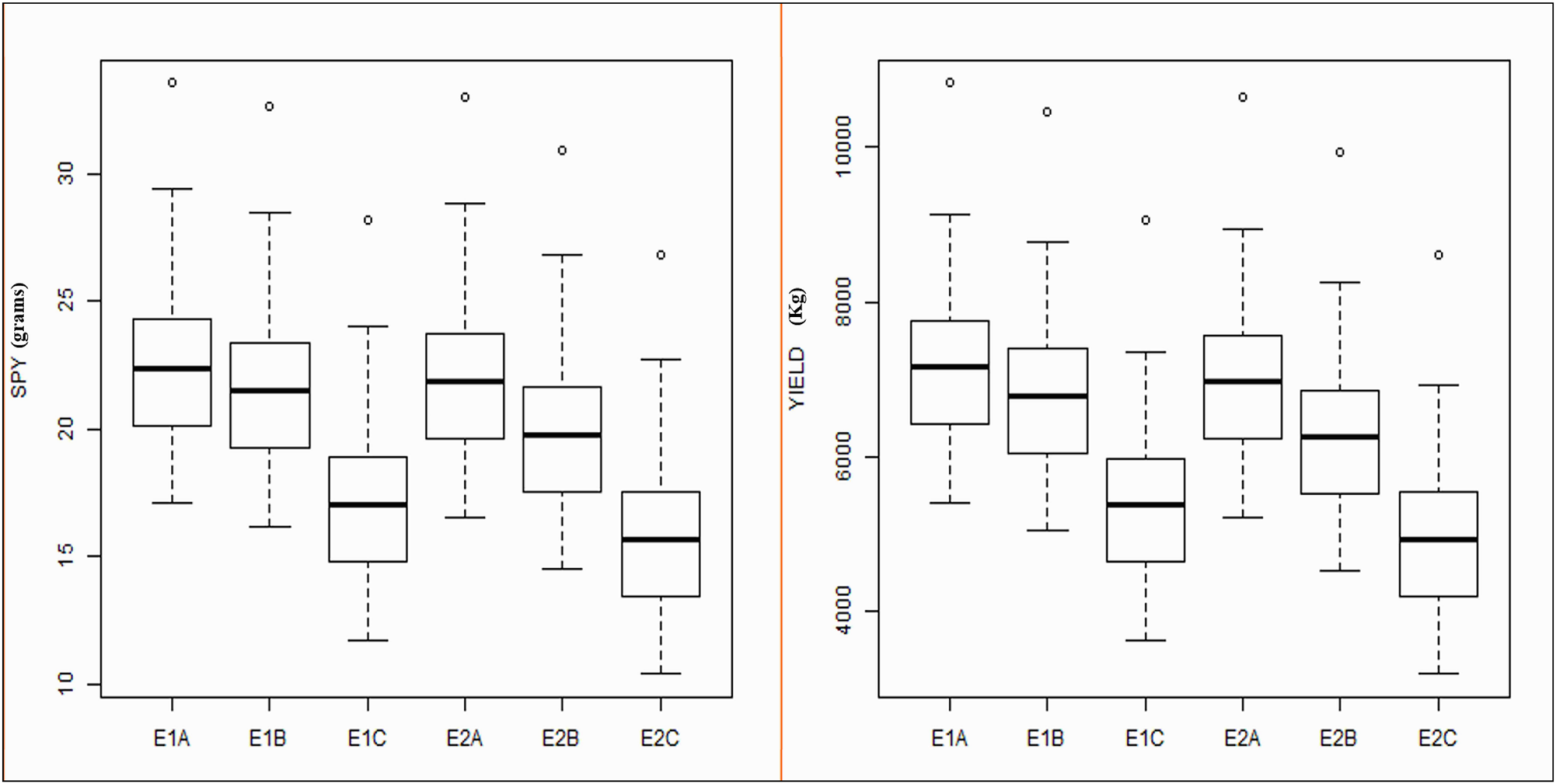
Box plot representation of genotypes performance for SPY and Yield traits across the environments.

DFF showed earliness in E1B and E2B environments; however, there was no significant difference observed for PH across the environments. For NRT, PL and TW considerable variation was observed for different sowing environments for the second year only. The PL and PW showed better performance in first set of sowing (E1A, E2A—No stress). The GP and SF showed reduction in E1B and E2B whereas SPY and YLD showed much reduction in performance in third set of sowing (E1C, E2C—Heat stress) during both the years (Fig. 1 and Supplementary Figure 1) indicating the existence of optimum environment for crop growth even with moderate temperature stress than high temperature stress. Similarly, a significant genotypic variation or expression as observed from higher phenotypic range was also observed in the second set of sowing (E1B and E2B) indicating that yield was not affected by moderate heat stress in that environment. Data of second and third set of sowing clearly exhibited reduction in yield levels compared to timely sowing condition.

**Figure 2:**
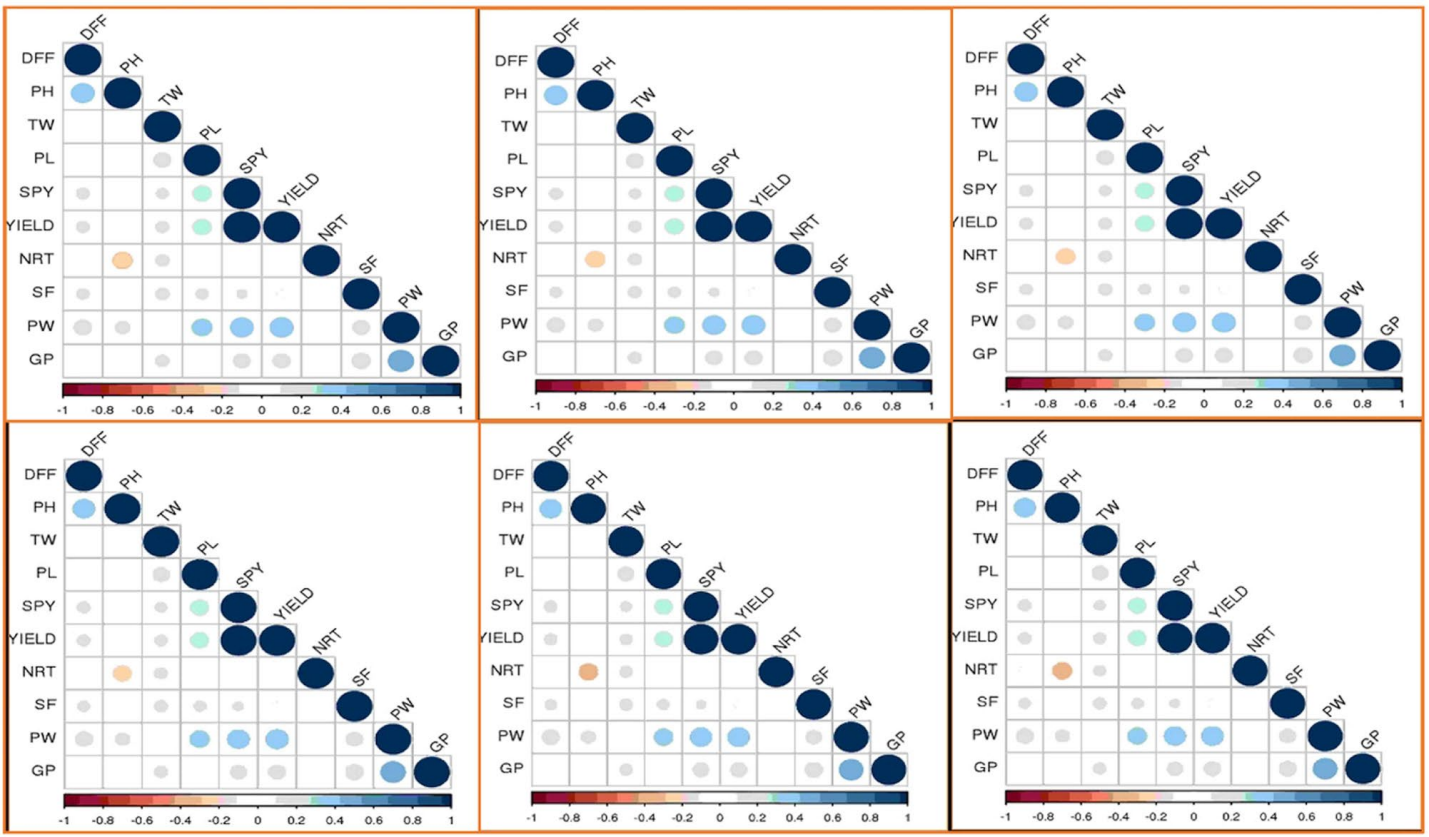
Phenotype correlation coefficient analysis using the Pearson method for all traits under study across the environments—E1A (Top Left), E1B (Top Middle), E1C (Top Right), E2A (Bottom Left), E2B (Bottom Middle), E2C (Bottom Right).

Correlations among the studied traits across the individual environments were represented in Fig. 2. Plot yield showed a significant trait correlation with all yield associated traits such as TW, PL, SPY, PW and GP across the six environments. Similarly, SPY showed a significant positive association with PL, PW and YLD. Among the six environments, GP has shown significant positive association with PW. In addition, PW also showed significant positive correlation with PL, SPY and Plot Yield.

### Genotypic adaptation and environment analysis

#### Stability analysis

From Finlay–Wilkinson analysis^15^, the genotypes viz., G54 (IR79156A × RPHR-1096), G21 (IR58025A × KMR3), G74 (IR58025B), G3 (APMS6A × KMR3) and G62 (IR68897A × 50–10) recorded regression value very close to zero exhibiting their yield stability.

#### AMMI Biplot analysis

**Figure 3:**
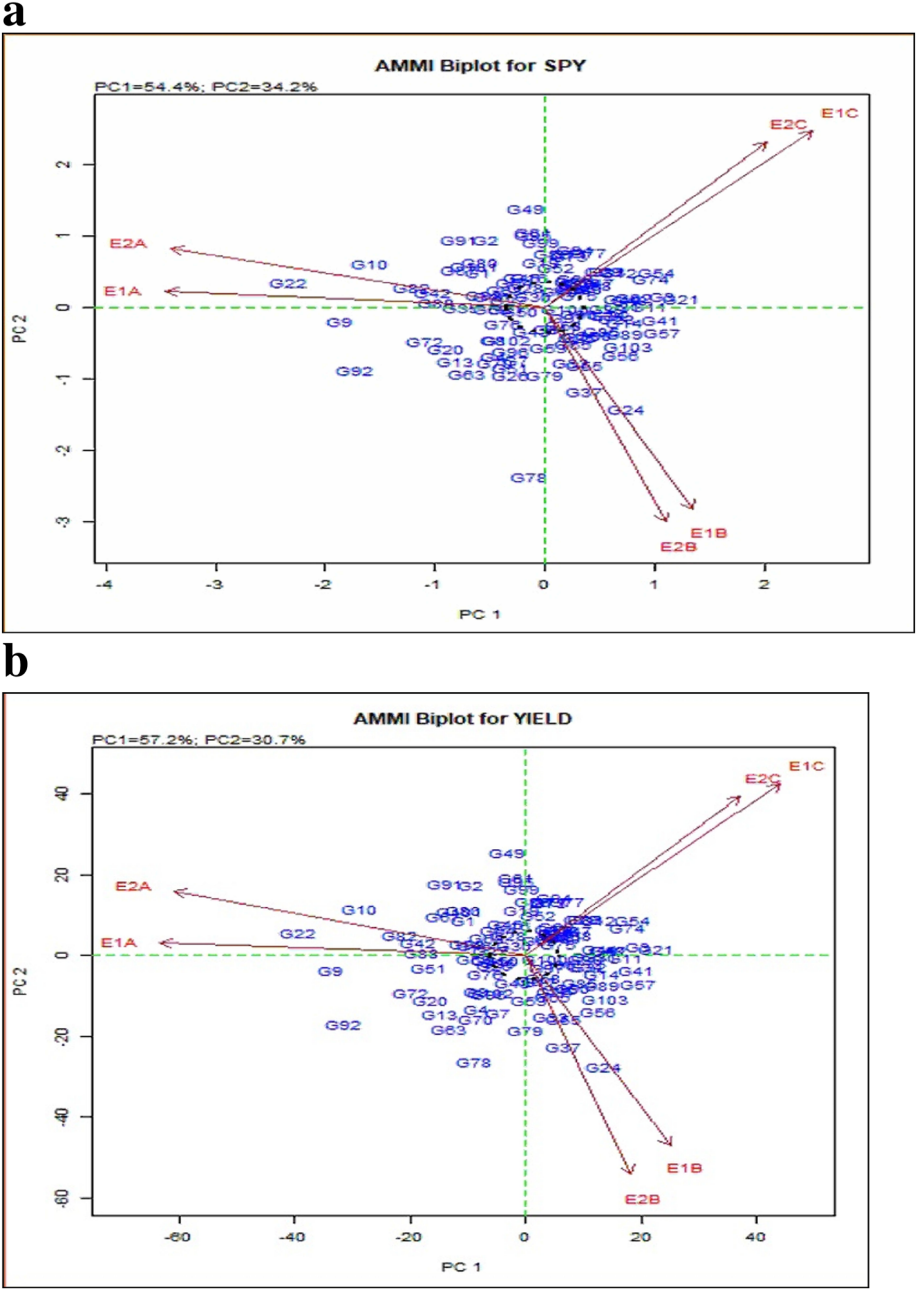

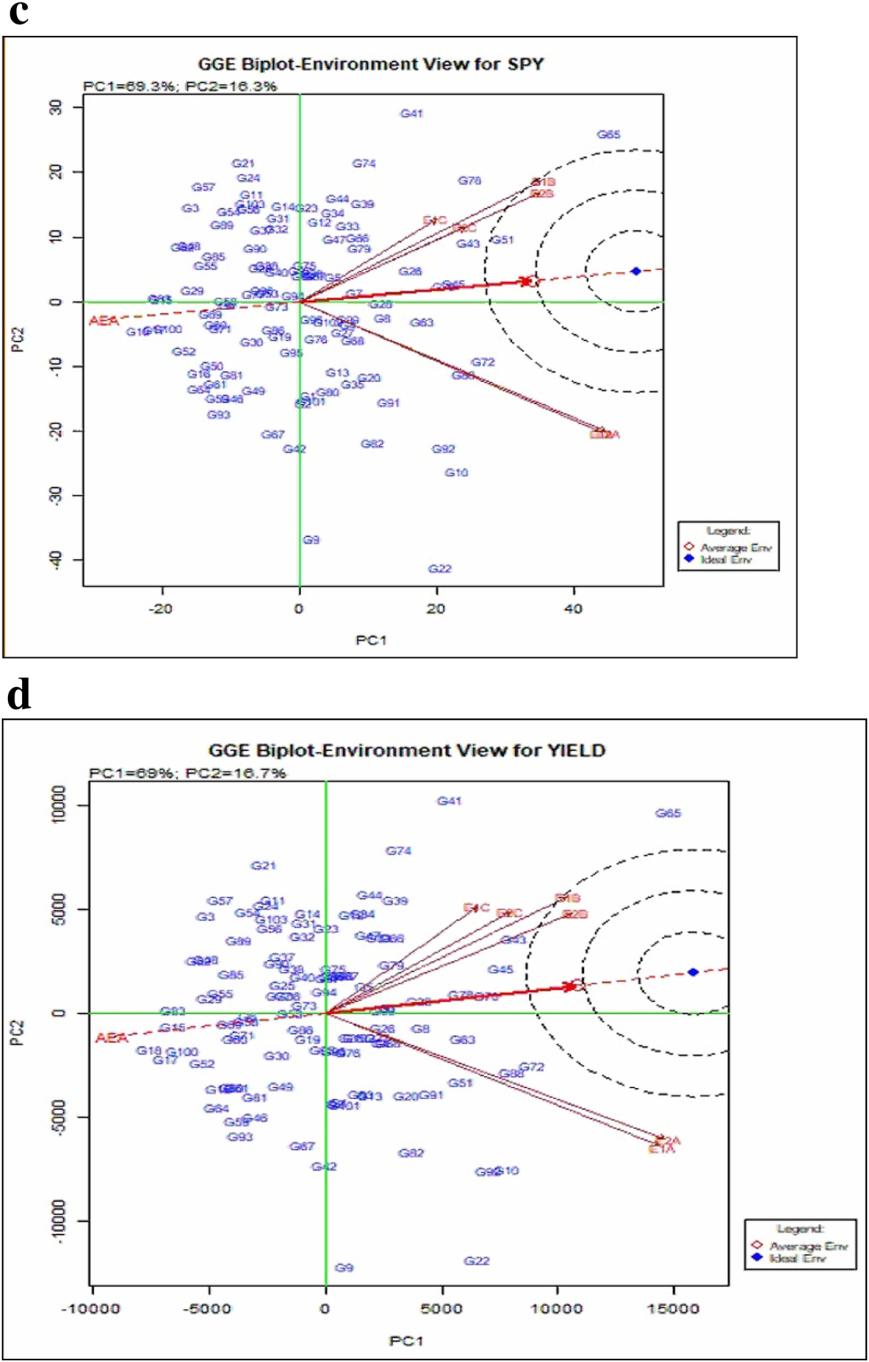
AMMI biplot for the primary component of interaction (PC1) and mean or main effect of rice genotypes in different environments showing relationship between environments and tested genotypes (For SPY (**a**), Yield (**b**)). GGE biplot for the primary component of interaction (PC1) and mean or main effect of rice genotypes in different environments showing relationship between environments and tested genotypes (For SPY (**c**), Yield (**d**)).

The analysis showed significant PC1 and PC2 components for most of the traits studied. The AMMI biplot indicated that most of the hybrids and their parental lines varying PC1 scores with mean grain yield of 6.29 t/ha (Fig. 3a, 3b and Supplementary Figure 2). Six environments differed from each other in both the years for main and interaction effects. The environment E1A and E2A had PC1 scores near zero and hence had small interaction effects, which indicated that all genotypes performed well in this environment and considered as the favourable environment for all the genotypes tested for SPY and YLD. The genotypes with zero score on the first PC were less affected by the interaction, while the genotypes with PC1 score close to zero and with above average yield were observed to show stability in yield levels with general adaptation to all the environments. A genotype with higher yield and PC1 had positive interaction showed that particular genotype is stable and adaptable to a specific environment. AMMI biplot showed a PC1 value of 54.4 and 57.2 for SPY and YLD, respectively. All the traits under study showed PC1 values above 50% and PC2 values above 20% except PL, which had a PC1 of 47% and PC2 of 24.7%. Hybrids such as G21 (IR58025A × KMR3), G3 (APMS6A × KMR3), G57 (IR68897A × KMR3) and G41 (IR79156A × RPHR1005) had average yield across environments and positive PC1 score indicating they are stable and favourably adapt to all environments or different sowing dates. G21 (IR58025A × KMR3) was also found stable for SPY; however, G88 (Akshyadhan) and G92 (IBL57) was more suited to stress tolerance due to their closeness to early and late sowing dates in both years with high yield and negative PC1 scores.

For DFF, G1 (APMS6A × BCW56) was found to be a more stable line in late duration, and the genotype G93 (BK 49–180) and G102 (Nagina22) was observed to be a stable line in the early crop duration group. Most of the genotypes clustered near to the second set of sowing environments, indicating their adaptability to normal sowing season. In GP, G65 (IR68897A × IR-66R) and G60 (IR68897A × RPHR517) had the highest trait values; however, hybrid G20 (IR58025A × EPLT104) and restorer line RPHR1005 were found to be more stable followed by G102 (Nagina22) and G35 (IR58025A × BK-49–180) which were more adapted to normal (E1A & E2A) and heat stress (E1C & E2C) environments. G69 (IR68897A × RPHR611-1), G71 (IR68897A × BK-49–180) and G70 (IR68897A × IBL57) were the most stable genotypes for NRT. However, most of the genotypes showed positive adaptation to moderate stress conditions. G12 (APMS6A × Akshyadhan) and G21 (IR58025A × KMR3) were stable genotypes with medium-tall stature and not influenced by high stress, while G69 (IR68897A × RPHR611-1) and G102 (Nagina22) showed stable SF trait. Highest PW was recorded in G72 (IR68897A × RPHR1096), whereas G92 (IBL57) was stable, while G23 (IR58025A × RPHR-1005) and G76 (IR68897B) expressed highest TW; However, G70 (IR68897A × IBL57) and G57 (IR68897A × KMR3) showed more stability for TW trait.

#### GGE analysis

GGE biplot analysis dissects the complex nature of GEI and simplifies them into various PC. According to GGE biplot environment view for yield (Fig. 3c and 3d), the environments E1A, E2A, E1B and E2B had a longer vector angle showing exertion of relatively strong interaction forces. The environment E1C and E2C had shorter vector angle and did not exert strong interactive forces. The specific adaptation to a target environment is not determined by the position and perpendicular projection of genotypes relative to environment vectors. If a genotype showing higher grain yield and the position of the genotype was further along the positive direction of an environment, they specifically adapted to that environment. Genotypic stability results of GGE were similar to that of AMMI biplot. Even though there was no severe high-temperature effect on plant establishment to the vegetative stage, the high temperature was most effective on the reproductive (grain filling) stage.

**Figure 4:**
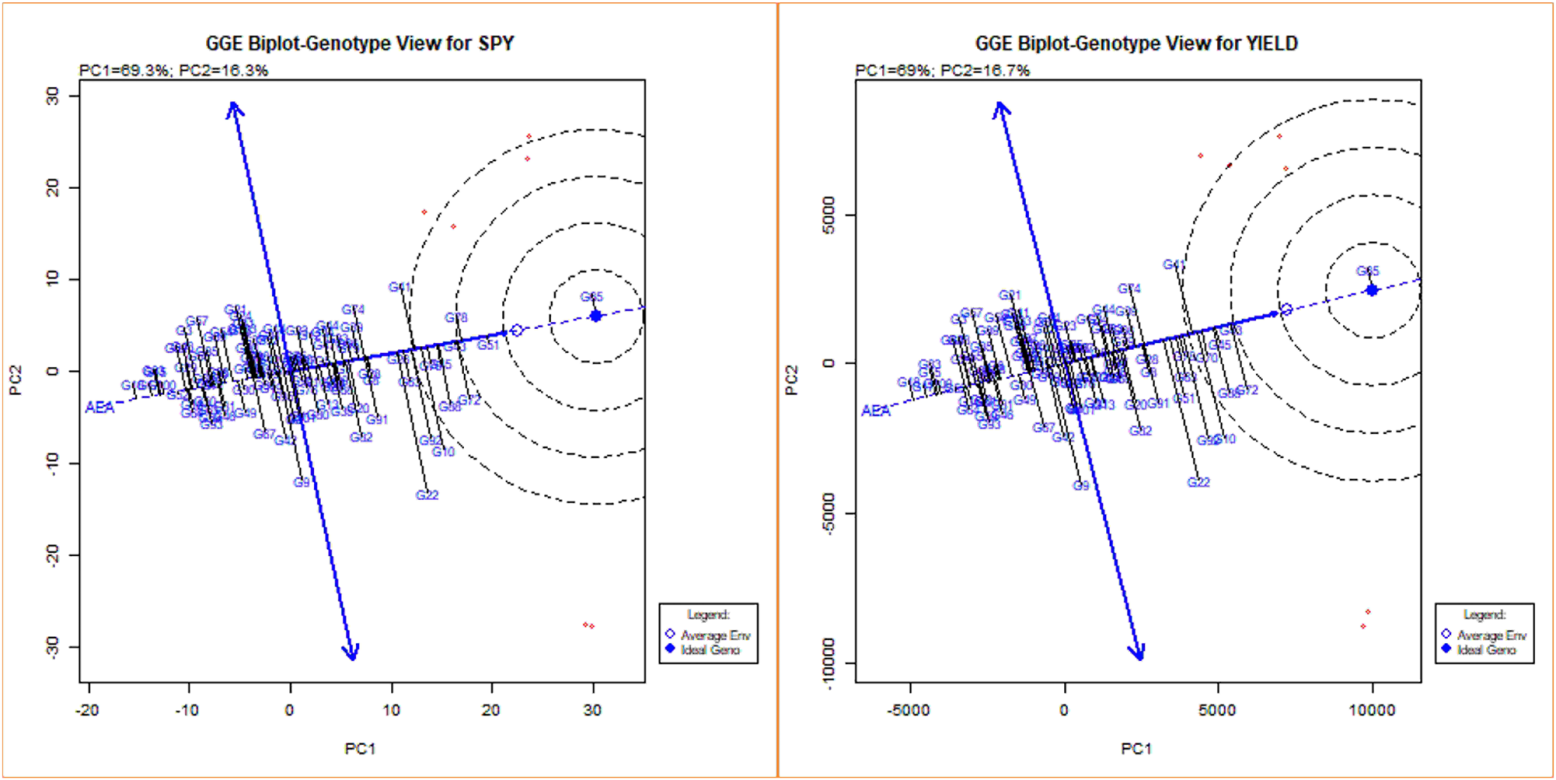
GGE biplot-Genotype view, including performance of test genotypes in comparison of to an estimated average environment and ideal genotype (For SPY and Yield Traits).

Figure 4 and Supplementary Figure 3 shows GGE biplot genotype view indicates that PC1 values accounted for more than 50% (69%) of total variability for all the traits under study and graphically represented PC1 and PC2 of the data set. In the case of DFF, ideal genotypes were not found in the late type, but G93 (BK 49-180) and G102 (Nagina22) was identified as mid-early and early stable line. The hybrids viz*.,* G65 (IR68897A × IR-66R) and G51 (IR79156A × RPHR611-1) were found closer to ideal and average environments for GP but were not stable as they were placed farther from Average Environment Axis (AEA). G78 (EPLT104), G100 (IR 64) and G50 (IR79156A × 363-5) were next in the order and closer to AEA and was observed to be more stable for the trait GP. For NRT, G58 (IR68897A × RPHR619-2) was found to show the highest value across the environments while G75 (IR79156B) and G3 (APMS6A × KMR3) were most stable. In the case of plant height, G12 (APMS6A × Akshyadhan) and G100 (IR64) were most stable, and the general trend of stability showing closeness to AEA was found in most of the genotypes for this trait. G1 (APM6A × BCW56) was found ideal for panicle length with the longest panicle followed by G29 (IR58025A × IR-66R), whereas G2 (APMS6A × EPLT104) was found most stable. G102 (Nagina22) was observed to have one of the shortest panicle lengths among the genotypes. For panicle weight, G73 (APMS6B) and G72 (IR68897A × RPHR1096) were most stable with the highest panicle weight as ideal genotypes for this trait. G67 (IR68897A × SG27-105) and G5 (APMS6A × RPHR1005) were found ideal genotype for SF and most of the genotypes centers around and found ideal for TW. Hybrid G18 (APMS6A × RPHR1096) and G17 (APMS6A × BK-49–180) were identified as stable and ideal genotype for SPY and YLD followed by G83 (IR40750R), G15 (APMS6A × RPHR611-1) and G100 (IR64).

**Figure 5:**
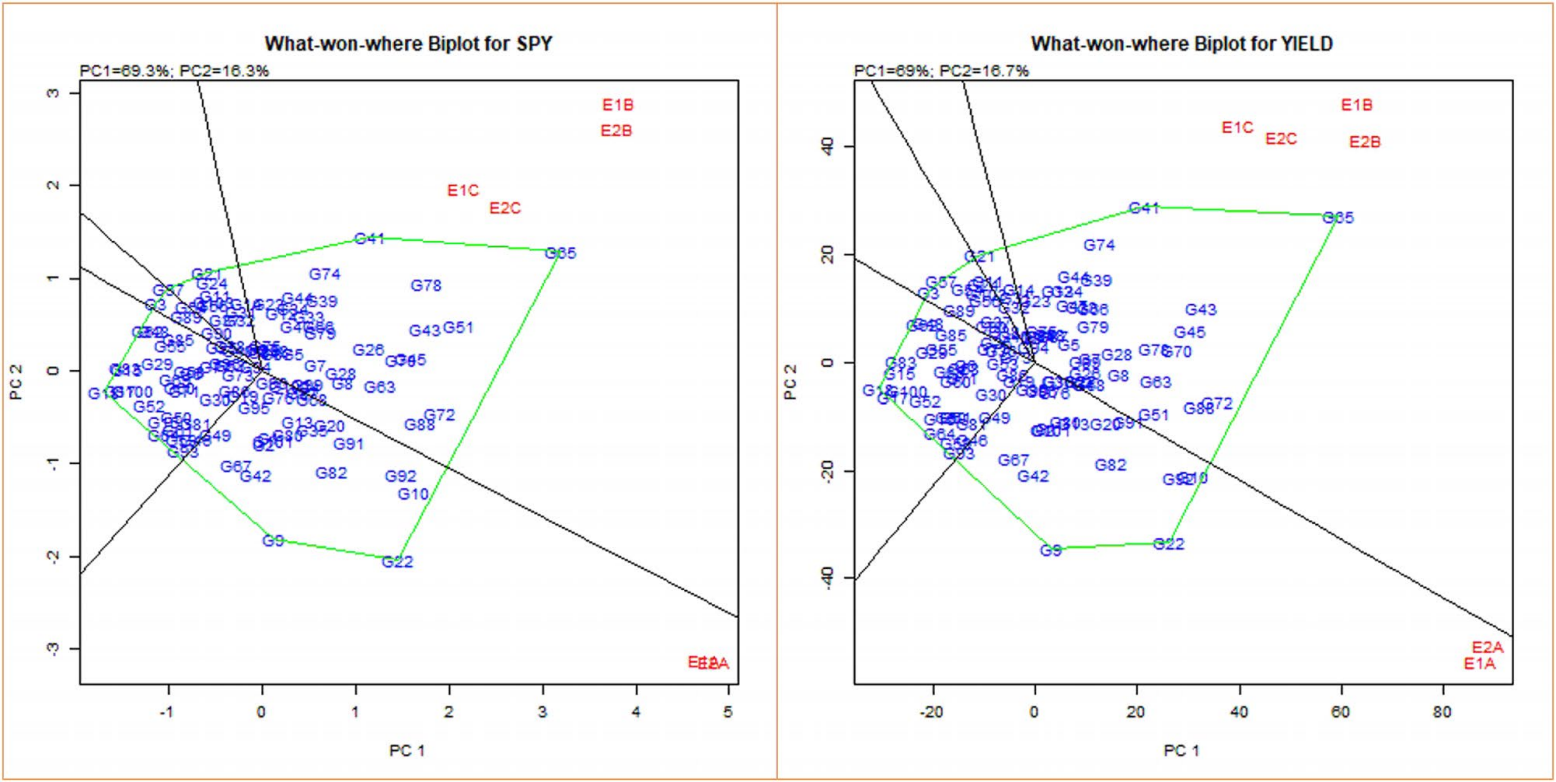
Polygon views of the GGE biplot based on symmetrical scaling for ‘which-won-where’ pattern of rice genotypes in six environments showing which genotype performed best in which environment (For SPY and Yield Traits).

Which-won-where polygon plots formed by linking the farthest genotypes from the biplot origin, including all the remaining genotypes in the polygon and those at the vertex, are the winning genotypes in the specific sector containing environments^16^. There were five sectors identified for, which won where the plot (Fig. 5 and Supplementary Figure 4). Cross of G65 (IR68897A × IR-66R) was the winner in the sector where the four environments were located and similarly G65 (IR68897A × IR-66R) was the vertex genotype for YLD where E1B, E2B, E1C and E2C were located.

**Figure 6:**
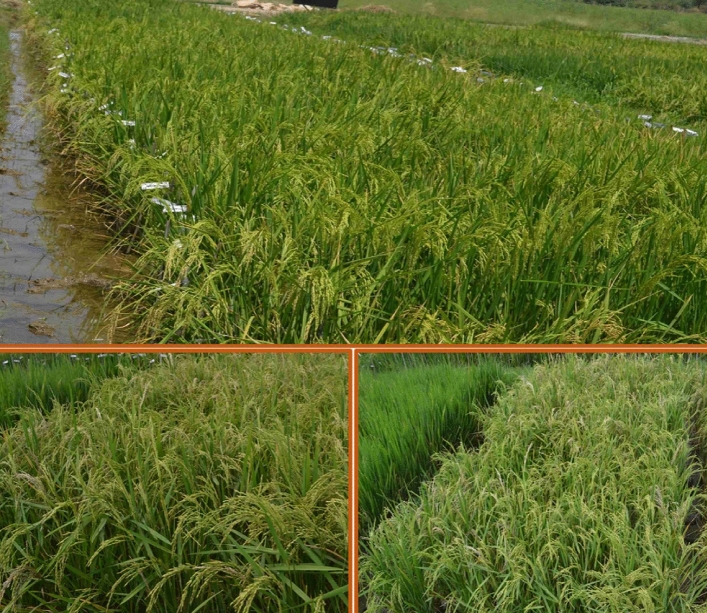
Overview of field at anthesis stage (E1C) during 2013–14 (Top); Hybrid (IR79156A × EPLT104) (E2C) during 2014–15 (Bottom left); Hybrid (IR58025A × 50-10) (E2C) during 2014–15 (Bottom right).

For DFF, three sowing dates placed in three distinct sectors, early environments E1A and E2A fell in the sector in which G83 (IR40750R) and G47 (IR79156A × IR-66R) were the vertex cultivars, showing they were the best-suited genotypes for normal sown environments E1A and E1B (Non-heat stress environment). Similarly, G11 (APMS6A × IR-66R) was suited for second sowing environments E1B and E2B (Moderate heat stress environment), and G79 (KMR3) was best suited for late sown environments E1C and E2C (High heat stress environment). For GP, G94 (RPHR1096) was best suited in E1A and E2B and remaining environments placed together and G60 (IR68897A × RPHR517), G65 (IR68897A × IR-66R) and G69 (IR68897A × RPHR611-1) were the most adapted genotypes across these environments. For NRT, G5 (APMS6A × RPHR1005) was the best suited in E1A, E2A; G10 (APMS6A × RPHR695-1) in E1B, E2B; G69 (IR68897A × RPHR611-1), G14 (APMS6A × 363–5) and G38 (IR79156A × EPLT104) in E1C, E2C. The field view of G38 (IR79156A × EPLT104) in E2C was represented in Fig. 6.

For PH, G101 (PA6444) and G23 (IR58025A × RPHR1005) was winners for E1C and E2C and G3 (APMS6A × KMR3) for the remaining four environments. G78 (EPLT104) and G43 (IR79156A × IR40750R) showed no change in PL in early and late sown environments. G73 (APMS6B) and G65 (IR68897A × IR-66R) were winners for PW in late sown environments. G72 (IR68897A × RPHR1096) for moderate stress and G96 (NDR359), G39 (IR79156A × KMR3) for early sown environments. G35 (IR58025A × BK-49–180), G41 (IR79156A × RPHR1005) and G78 (EPLT104) were the vertex genotypes for stress environments (E1B, E2B and E1C, E2C) respectively. For TW, G57 (IR68897A × KMR3) and G59 (IR68897A × RPHR1005) was the vertex genotype in which won where polygon harbouring E1A, E2A and E1C, E2C, whereas G23 (IR58025A × RPHR1005) was were adapted to moderate stress environments.

## Discussion

The primary constraint to hybrid rice cultivation and seed production during the dry season is elevated temperature, specifically during the reproductive stage, severely affecting spikelet fertility and eventually, grain yield. Therefore, the development of hybrids with higher yield heterosis coupled with stability at varying temperature levels should be the long-term objective of the modern breeding program. In the present study, variance analysis revealed significant GE difference for yield traits. GEI on phenotypic expression was observed due to change in the sowing date, causing different temperature regimes throughout the crop growth and development period. The performance of genotypes under varying temperature regimes was different. It was observed that PL was having high environmental influence on phenotypic expression. The percentage of explanation of phenotype by genotypic contribution was high for PH, DFF, GP, TW, and SPY, while genotype × environment interaction effect was high for yield, PW, and SF. DFF, PW, SPY and Yield showed a similar trend in phenotypic variability across 3 temperature regimes in both the seasons. In case of SPY and yield, the reduction in yield according to increase in temperature stress was very obvious for all the genotypes under the study. NRT, PL, PW and SF also showed the reduction in trait values in both moderate and severe heat stress compared to normal temperature condition. Traits like PH, GP and TW showed the variation due to environment with different temperature regimes only in second season.

Genotype and environment interactions were found to be significant in the pooled analysis for all the traits. Given the higher spikelet number, hybrids having a substantial yield advantage over conventional cultivars (IR64) at 29 °C to 35 °C; however, advantage fades away when the temperature shoots beyond 38 °C^17^. The AMMI, GGE biplots provides information on interaction of genotypes and environments, the AMMI model^18^ collectively considers environment (E), genotype (G), and their interaction with each other (GEI) as an individual parameter for the evaluation purpose, whereas the GGE biplot^19^ evaluate the interaction by considering the genotype (G) and genotype’s environmental interaction (GE). The PC scores and ASV values reveal more information on variation among genotypes^20^. The AMMI biplot for SPY and YLD indicated that most genotypes inclined to have PC1 scores of nearly zero, and their mean grain yield was close to around 600–700 g m^−2^. The environments differed from each other not only for the main effect but also for the interaction effect. The environments E1A/B and E2A/B had PC1 scores near zero for SPY and hence with small interaction effects, indicating that most of the genotypes performed well in these two early sown environments. Thus, these two environments can be considered favourable environments for all the genotypes tested for SPY.

Similarly, those genotypes with zero score for any trait on the first PC1 were less influenced by the environments. Furthermore, those lines with above average yield and PC1 score close to zero were considered stable and had a general adaptation to all the environments. The genotypes located near to the ideal genotypes in GGE biplots were showing higher trait values. However, the distance from AEA determines their stability. Similar kinds of observations were reported earlier^21–25^.

AMMI and GGE generally clustered the early (E1A, E2A), mid (E1B, E2B) and late (E1C, E2C) sowing environments in both the years separately for DFF, GP, NRT, PL, PW, SF, SPY, TW and yield. While DFF for E2C, GP and PL for E2B, SPY for E2A and PH and NRT for E1A, E2B were the environments placed in negative PC1 axis. Biplots are useful in determining the significant relationship of cropping season and weather parameters on grain yield, contributing to the GEI^26^. The conclusion on the relationship between the testing environments can be estimated based on the angle between their vectors. The present study indicated that the environments were related based on their sowing time and further temperature regime variations in the cropping season. The discriminating ability of environments on genotypes was interpreted from GGE biplots environment view indicated from projections of the environment vectors concerning the concentric circles^19^. Thus late environments, E1C, and E2C with lower vector length, were less discriminating than medium and early sowing environments in case of yield. This is mainly due to the general yield decline in most of the genotypes in late sown as they were subjected to severe temperature stress. Wide obtuse angles between environment vectors in case DFF, NRT, PL, SPY and YLD showed strong negative correlation among the testing environments suggesting the presence of strong crossover GE. This indicates that the genotypes performing better in one environment might be performing poorly in other environments suggesting the specific adaptability of genotypes. The existence of crossover and non-crossover GEI in multi-environment testing is widespread, especially in multi-location trials^27^. Which-won-where graphs of GGE biplot address different factors like crossover GE, mega-environment differentiation and specific adaptation^24,28–30^. Based on GGE biplot analysis, the testing environments were classified into three mega-environments, comprising early (E1A and E2A), medium (E1B and E2B) and late (E1C and E2C).

In this study, it was observed that restorer lines are comparatively more tolerant to high-temperature stress than the maintainer lines. Hence male parent contributes more to the tolerance under elevated temperature. These results were similar to the earlier reports^11^, where the heat stress index of F_1_ combinations was significantly correlated with the heat stress index of restorer lines but not with the heat stress index of maintainer lines. The *indica* hybrid rice combinations with pureline *indica* male parent showed higher heat tolerance than those with permeability from *japonica* male parent^8^. Moreover, it was identified that the hybrid IR82378H performed well under severe drought conditions than in well-watered conditions and could be due to the effect contributed by the male parent^14^. The heat tolerant hybrids derived from the heat-susceptible × heat-tolerant combination can be useful to identify the major QTLs governing heat tolerance in rice. However, the interaction of some minor genes with the QTLs, maternal inheritance may play a vital role in modifying effect as the hybrid combinations showed a moderate level of tolerance. This could also be attributed to the buffering capacity of hybrids to stress conditions. The study inferred that hybrids are generally tolerant to stress and exploitation of heterosis depends on yield per se and combining ability of parental lines.

There was no correlation between the duration of the genotype and the level of tolerance to high temperature stress. Some early maturing and few late maturing genotypes and hybrids showed tolerance to increased temperature. In farmer’s field conditions, early maturing genotypes have an advantage of escape from heat stress, and mostly their critical growth and development stages will be under minimal heat stress.

The genotypes under the present investigation were grouped into stable and adaptable across the environments for each trait. The AEC abscissa (AEA) is higher mean trait value indicator across environments. Thus, the hybrid G65 (IR68897A × IR-66R) had the highest mean yield and SPY with better stability, followed by hybrids G21 (IR58025A × KMR3), G3 (APMS6A × KMR3), G57 (IR68897A × KMR3) and G41 (IR79156A × RPHR1005) in terms of both yield and stability and are good candidates for cultivation across different environments (Supplementary Tables 1 and 2). Rice is adapted to a wide range of environmental conditions in India with varying sowing dates and temperature regimes and these stable genotypes are expected to be suitable across extensive range of environmental conditions.

## Conclusion

To maximize the yield potential of hybrids, it is imperative to select stable parental lines adaptable to suitable locations with favourable temperature and relative humidity with minimal risk from climatic conditions. In India, dry (*Rabi)* season is mostly affected by high-temperature stress at the reproductive stage, especially in April and May. The selection of early genotypes with high yield potential to the target environments and early sowing would be useful to minimize yield losses due to high-temperature stress. By analysing the effects G × E interaction on rice yield under different temperature regimes, some promising hybrids viz., G21 (IR58025A × KMR3), G3 (APMS6A × KMR3), G57 (IR68897A × KMR3) and G41 (IR79156A × RPHR1005) were identified for cultivation in the different temperature regimes whereas G65 (IR68897A × IR-66R), G74 (IR58025B), G83 (IR40750R), G85 (C20R) were found to be more adapted and perform better in higher temperature regimes whereas hybrids G18 (APMS6A × RPHR1096), G17 (APMS6A × BK-49–180) and G100 (IR64) expressed higher yield potential in favourable non-stress environments.

The stability analysis involving hybrids and parental lines from breeding programmes for irrigated ecosystems across three testing environments, in the present study, indicated the significant role of GEI for grain yield under different temperature regimes. Further investigation on the mechanisms of temperature tolerance in terms of physiological and molecular parameters for identifying the better genotype for grain yield is essential. The identified tolerant genotypes can be utilized further in the crop improvement programme.

## Material and methods

### Experiment particulars

The experimental material comprises of 103 genotypes which includes 72 rice hybrids, 18 restorer lines, four cytoplasmic male sterile (CMS) lines and nine varietal & hybrid checks (Supplementary Table 3). The experiment was conducted under three temperature regimes accomplished through three different dates of sowing with 15 days intervals over two dry seasons (2013–14 and 2014–15). Each sowing date is expected to result in a different environmental condition with varying temperatures across the crop growth stages. Environmental conditions during the crop growth period are presented in Supplementary Figure 5a and 5b. Three temperature regimes consist of six environments over two years (E1A, E1B and E1C in 2013–14; E2A, E2B, and E2C in 2014–15).

**i) Ambient temperature regime**—E1A (DS: 09-Dec-2013) and E2A (DS: 10-Dec-2014) considered as a control condition (timely sown) and the flowering period of crop escapes with no temperature stress (~ during the second fortnight of March).

**ii) Moderate temperature stress regime**—E1B (DS: 24-Dec-2013) and E2B (DS: 26-Dec-2014) belonging to mid-late sowing (over 15 days after sowing the first set) and the flowering period of crop matches with moderate temperature stress (~ during the first fortnight of April).

**iii) High-temperature stress regime**—E1C (DS: 08-Jan-2014) and E2C (DS: 10-Jan-2015) belonging to late sowing (over 15 days after the sowing of the second set) and the flowering period of crop matches with high-temperature stress (~ during the second fortnight of April).

The experiment was carried out under irrigated conditions at Research Farm of ICAR-Indian Institute of Rice Research (IIRR), Hyderabad, India, located at 17° 19′ N and 78° 29′ E and an altitude of 549 m above mean sea level. The farm soil is alkaline vertisol, with a pH of 7.94. Before sowing, the seeds were treated with Carbendazim 50% WP (Wettable Powder) and allowed to germinate in petri dishes layered with blotting paper. Germination rate were recorded and after five days, the pre-germinated seeds were transferred to wet nursery bed topping with a layer of vermicompost and sand. The top dressing was applied before one week of uprooting with Di-Ammonium Phosphate (DAP) @40 kg ha^−1^ and need-based plant protection measures were undertaken to raise healthy and vigorous seedlings. Twenty-seven days old healthy seedlings were transplanted to the main field in randomized complete block design (RCBD) with two replications of 10 m^2^ with 20 cm spacing between rows, 15 cm between plants within row and no vacant row was left between two genotypes. The recommended dose of fertilizers 100 kg ha^−1^ of Nitrogen in three split doses with one fourth as basal dose, one half at the time of initial tillering stage and one fourth at active tillering stage, entire dose of 40 kg ha^−1^ of phosphorus and 60 kg ha^−1^ of Muriate of Potash (MOP) per hectare were applied at the time of transplanting as basal dose. Required weed management and plant protection measures were timely undertaken for healthy crop production.

### Measurement of grain yield and component traits

A total of 103 genotypes were phenotyped for different yield and yield-related traits. Days to 50% flowering (DFF) was measured on plot basis. Data on plant height (PH), number of productive tillers (NRT), panicle length (PL), panicle weight (PW), number of grains per panicle (GP), spikelet fertility (SF), thousand seed weight (TW), single plant yield (SPY) and grain yield per plot (YLD) were recorded at maturity. The data on above traits were recorded in all the entries using modified Standard Evaluation System for Rice (SES)^31^ at flowering and maturity stages respectively.

### Statistical analysis

Replicated phenotypic data of the agro-morphological and yield traits across six environments were subjected to the combined analysis of variance (ANOVA) using PB Tools ver.5 statistical software (http://bbi.irri.org/products) (Supplementary Table 4). Correlation coefficients were estimated according to the Karl Pearson method^32^. In this study multivariate model such as AMMI biplot and GGE biplot was used to determine the genotype and environment interaction and its relationship with stability parameters using PB Tools ver.5 statistical software (http://bbi.irri.org/products) and R^33^. The performance of hybrids and corresponding parental lines were tested over two seasons and assessed using stability models for G × E and yield stability analyses based on the principal component analysis (PCA): viz., Additive main effects and multiplicative interaction (AMMI)^34^ and GGE Biplot or site regression model^35^. In AMMI, the GEI effects are presented by genotype and environment effects plotted in a biplot whereas in GGE, the genotype and GEI effect using environment-centered PCA were presented. ANOVA is used to estimate main effects while PCA decomposes the interaction into PCA axes. The AMMI model separates the additive variance from multiplicative variance. The AMMI stability value (ASV) was calculated (Purchase et al. 2000) and PCA is required to study the genotype and environment interaction component. ANOVA, which is an additive model, is effective in apportioning the total sum of squares into genotype main effect, environment main effect and GE interaction, but does not provide insight into the GEI structure.

The analytical model can be written as

$$Y_{ij.} = \mu + \delta_{i} + \beta_{j} + \sum\limits_{k = 1}^{K} {\lambda_{k} \delta_{ik} \beta_{jk} + \varepsilon_{ij.} }$$

Both G and GE variation were graphically represented by GGE biplots^36^ using sites regression (SREG) linear–bilinear model as given in the formula

$$Y_{ij.} = \mu + \beta_{j} + \sum\limits_{k = 1}^{K} {\lambda_{k} \delta_{ik} \beta_{jk} + \varepsilon_{ij.} }$$

where, *Y*_*ij*_.—mean yield of *i*th genotype in *j*th environment, μ—Overall mean, δ_*i*_—genotypic effect, β_*j*_ -environment effect, λ_*k*_—singular value for PC axis k, δ*ik*—genotype eigenvector value for PC axis n, β_*jk*_—environment eigenvector value for PC axis k, ε_*ij*_—residual error assumed to be normally and independently distributed (0, σ2/r), σ2—pooled error variance and r is the number of replicates.

### Ethical approval

All the experiments carried out on plants were carried out in accordance with the guidelines of ICAR – Indian Institute of Rice Research.

## Supplementary information

Supplementary Information.

## Abbreviations

AMMI: Additive main effect and multiplicative interaction
PCA: Principal component analysis
GGE: Genotype and genotype × environment interaction
GEI: Genotype environment interaction
IIRR: Indian Institute of Rice Research
DS: Date of sowing
DFF: Days to fifty percent flowering
PH: Plant height
GY: Grain yield
SF: Spikelet fertility
PL: Panicle length
PW: Panicle weight
NRT: Number of productive tiller
GP: Grains per panicle
TW: 1000 seed weight
SPY: Single plant yield
YLD: Plot yield
