# Supplementary material for: Evaluation of genotype by environment interaction and adaptability in lowland irrigated rice hybrids for grain yield under high temperature
Source: Sci Rep. 2021 Aug 4;11:15825. doi: 10.1038/s41598-021-95264-4 (PMC8338964; doi:10.1038/s41598-021-95264-4)
Supplement: Supplementary file 1 — Supplementary Information. [file 41598_2021_95264_MOESM1_ESM.pdf]

Evaluation of Genotype by Environment interaction and Adaptability in lowland irrigated rice hybrids for grain yield under high temperature

Senguttuvel P[1]*, Sravanraju N[1], Jaldhani V[1], Divya B[1], Beulah P[1], Nagaraju P[1], Manasa Y[1], Hari Prasad AS[1], Brajendra P[1], Gireesh C[1], Anantha MS[1], Suneetha K[1], Sundaram RM[1], Sheshu Madhav M[1], Tuti MD[1], Subbarao LV[1], Neeraja CN[1], Bhadana VP[2], Rao PR[1], Voleti SR[1], Subrahmanyam D[1]

1. Crop Improvement Section, ICAR-Indian Institute of Rice Research, Hyderabad-500030, India

2. ICAR - Indian Institute of Agricultural Biotechnology, Ranchi-834010, India

*****Dr. P. Senguttuvel (email: senguttuvel@gmail.com), Hybrid rice division, Crop Improvement Section, ICAR-Indian Institute of Rice Research, Hyderabad-500030, India +91 40 24591297; Fax: +91 40 24591217

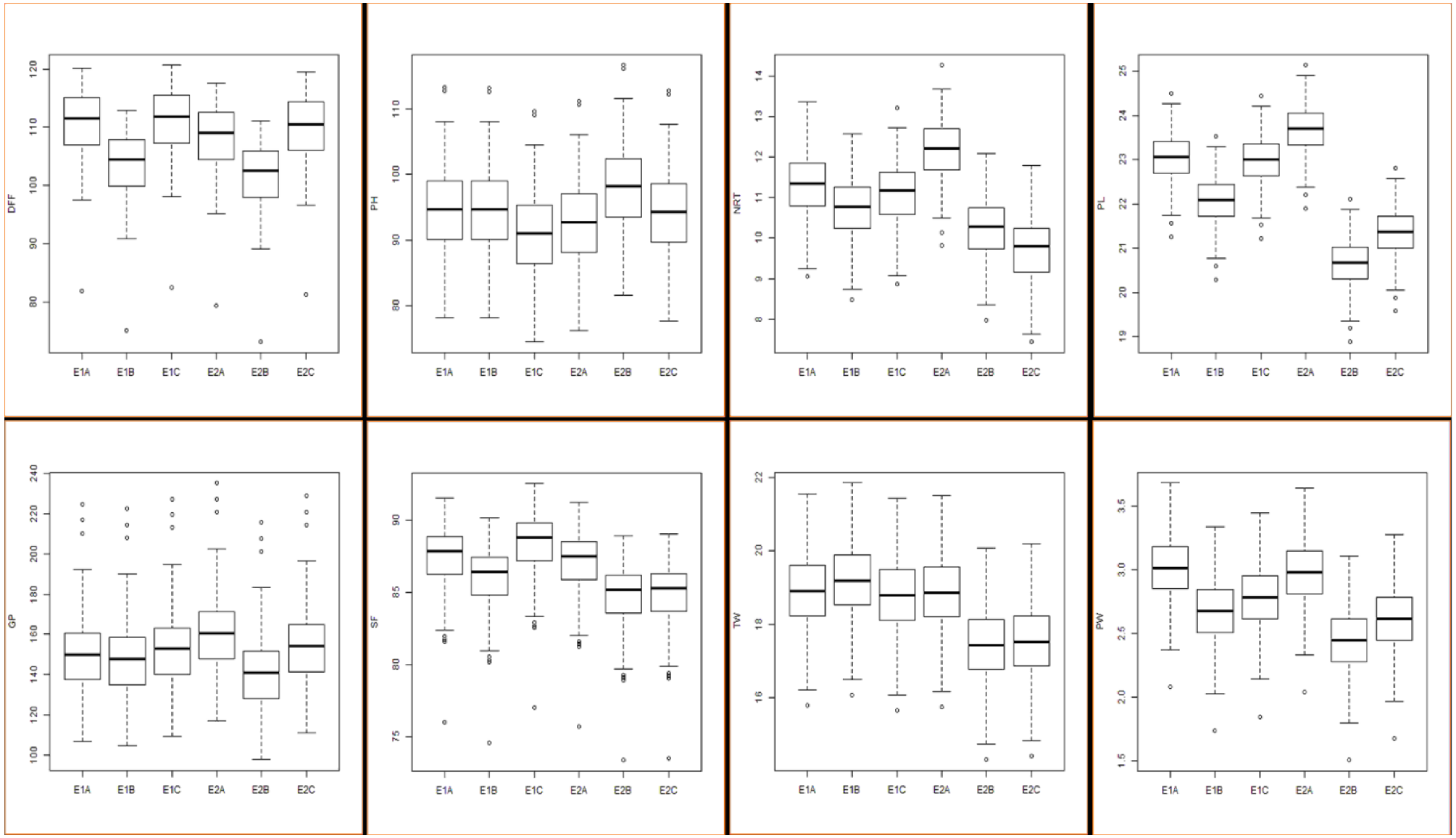

Supplementary Figure 1. Box plot representation of genotypes performance across the environments (Other than Yield and SPY traits)

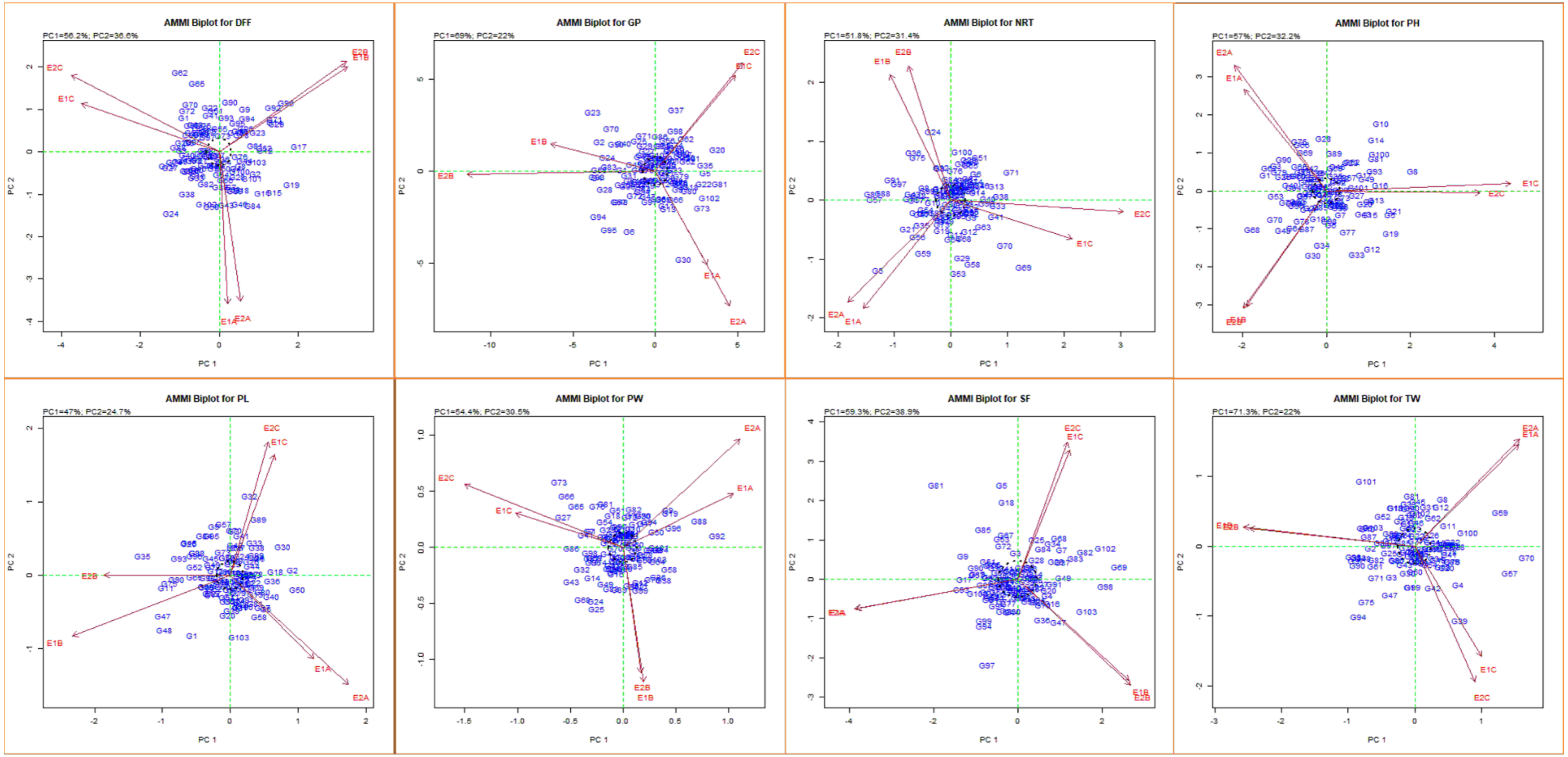

Supplementary Figure 2. AMMI and GGE biplot for the primary component of interaction (PC1) and mean or main effect of rice genotypes in different environments showing relationship between environments and tested genotypes (Other than SPY and Yield Traits).

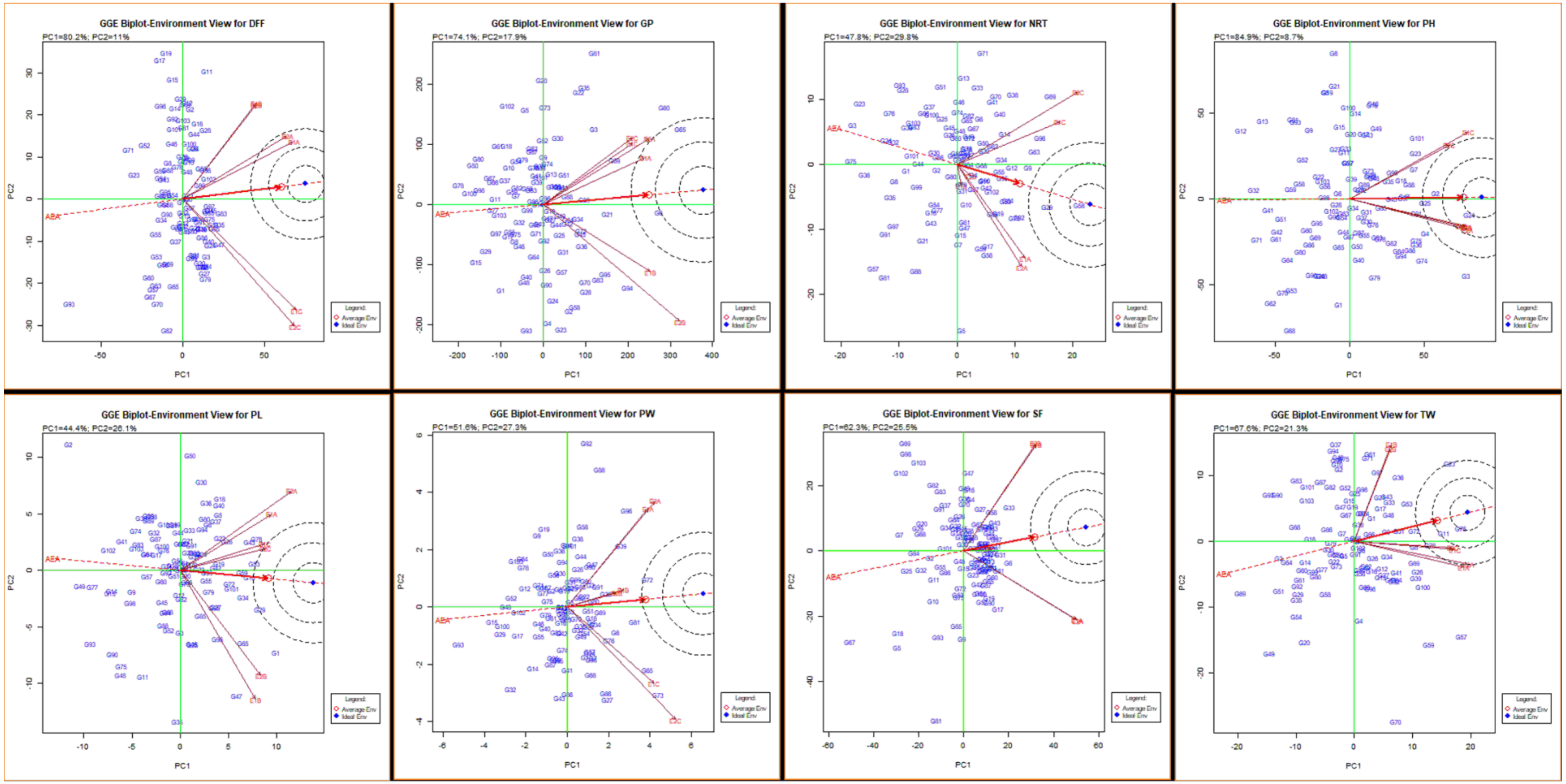

Supplementary Figure 3. GGE biplot-Genotype view, including performance of test genotypes in comparison of to an estimated average environment and ideal genotype (Other than SPY and Yield Traits).

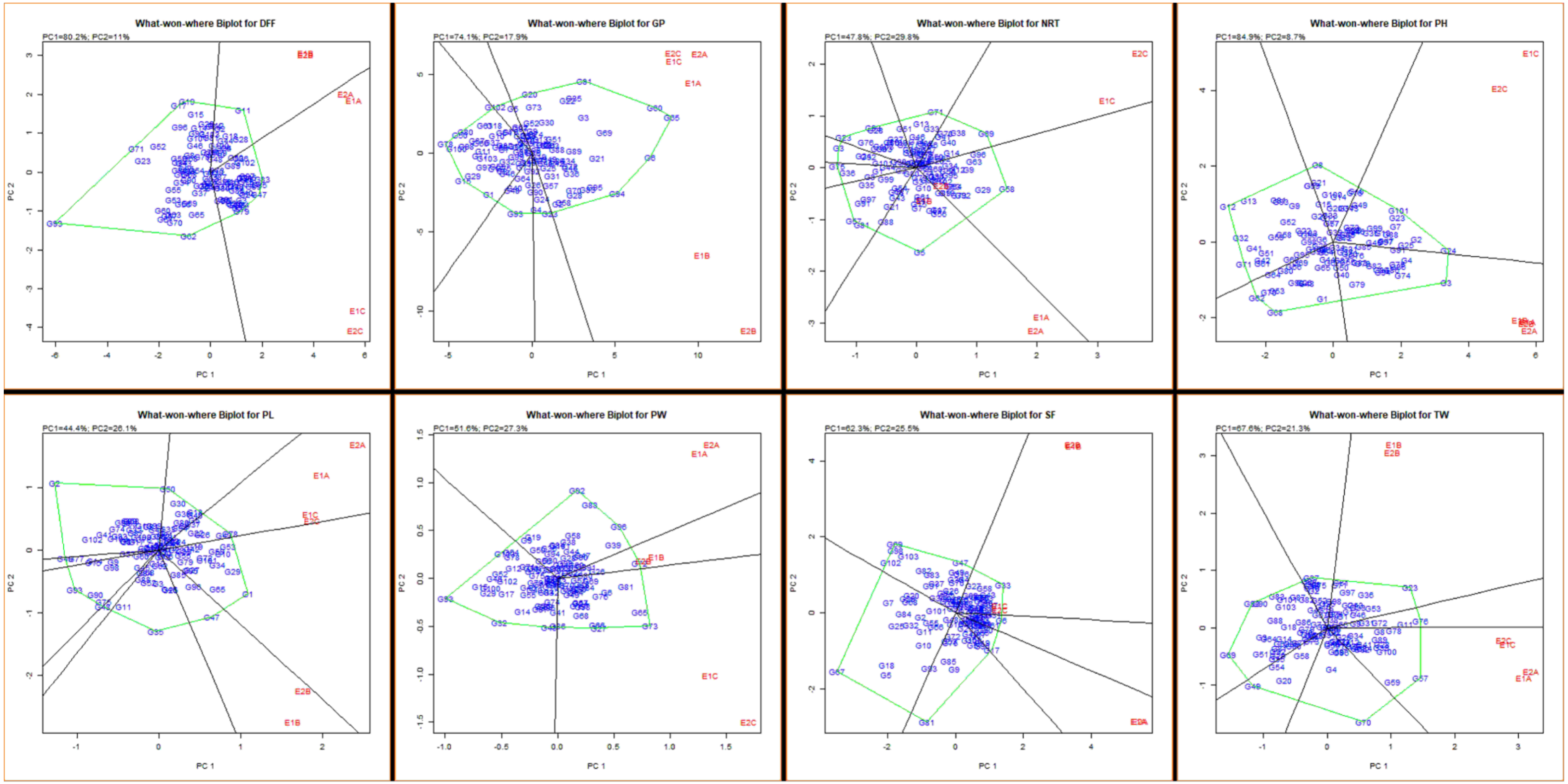

Supplementary Figure 4. Polygon views of the GGE biplot based on symmetrical scaling for 'which-won-where' pattern of rice genotypes in six environments showing which genotype performed best in which environment (Other than SPY and Yield Traits).

Season	*Dry season 2013-14*		
**Temperature regimes**	**Control**	**Moderate Temperature Stress**	**High Temperature Stress**
**Environments**	**E1A**	**E1B**	**E1C**
**Sowing dates**	17-Dec-2013	25-Dec-2013	02-Jan-2014
**Transplanting**	22-Jan-2014	24-Jan-2014	03-Feb-2014

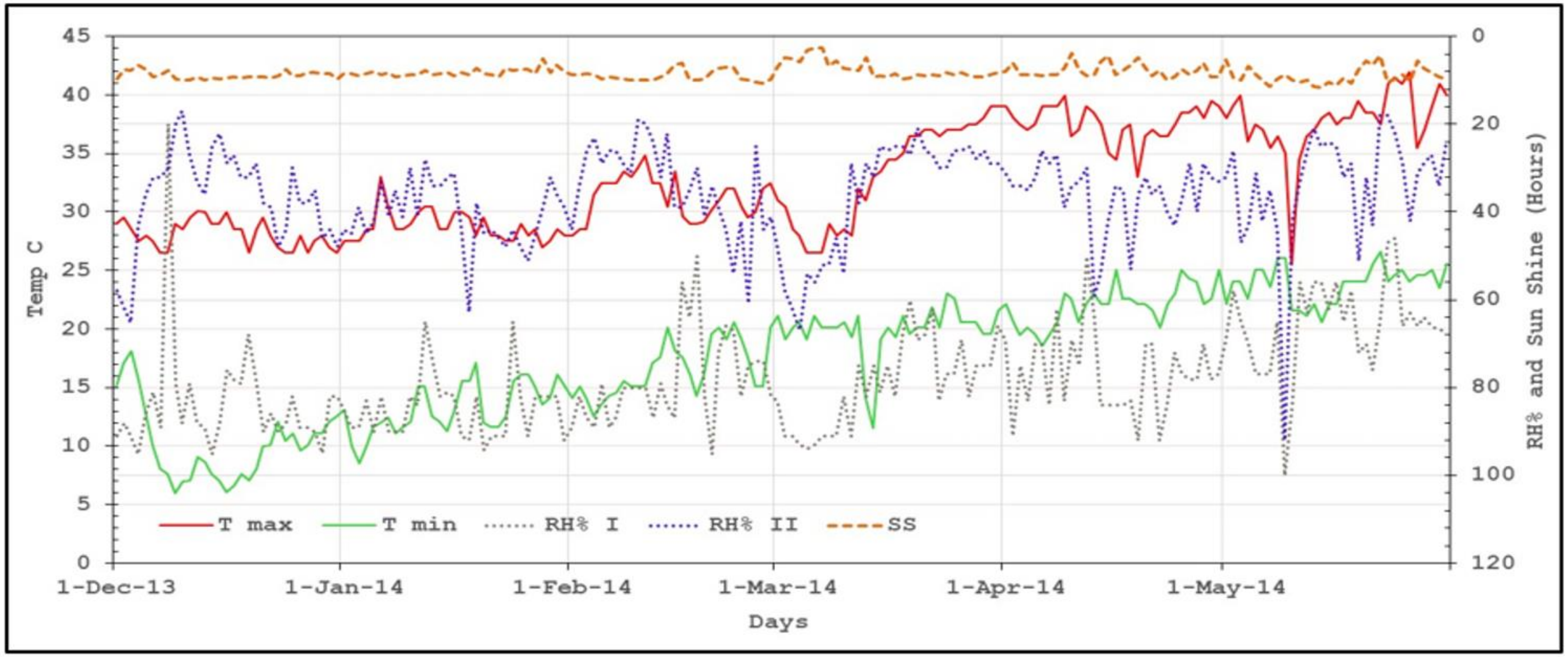

**E1A**	Sowing	Seedling establishment	Reproductive	Vegetative	Maturation	-	-
**E1B**	-	Sowing	Seedling establishment	Reproductive	Vegetative	Maturation	-
**E1C**	-	-	Sowing	Seedling establishment	Reproductive	Vegetative	Maturation

Supplementary Figure 5a. Temperature and other weather parameters during crop season 2013-2014.

**Season**	***Dry season 2014-15***		
**Temperature regimes**	**Control**	**Moderate Temperature Stress**	**High Temperature Stress**
**Environments**	**E2A**	**E2B**	**E2C**
**Sowing dates**	18-Dec-2014	26-Dec-2014	03-Jan-2015
**Transplanting**	23-Jan-2015	29-Jan-2015	07-Feb-2015

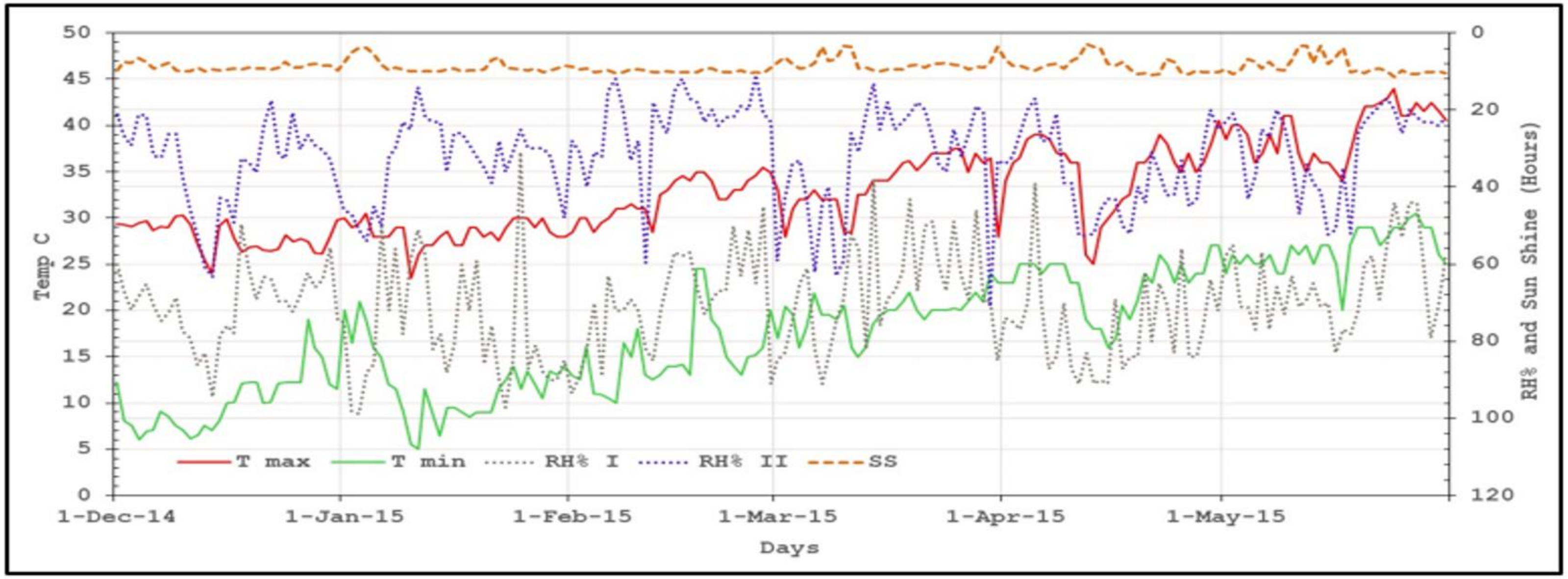

**E2A**	Sowing	Seedling establishment	Reproductive	Vegetative	Maturation	-	-
**E2B**	-	Sowing	Seedling establishment	Reproductive	Vegetative	Maturation	-
**E2C**	-	-	Sowing	Seedling establishment	Reproductive	Vegetative	Maturation

Supplementary Figure 5b. Temperature and other weather parameters during crop season 2014-2015.

Supplementary Table 1. The mean data of most stable Parental lines Hybrids (along with checks) across the environments in the current study.

Code	Designation	ENV	DFF	PH	NRT	PL	PW	GP	SF	TW	SPY	YIELD
G3	APMS6A × KMR3	E1A	116	113.53	8	23.12	3.27	184	89.91	16.35	18.89	5992.86
G3	APMS6A × KMR3	E1B	109	113.35	8	22.15	2.93	182	88.48	16.64	17.96	5635.10
G3	APMS6A × KMR3	E1C	117	109.57	8	23.07	3.04	186	90.87	16.22	13.48	4217.10
G3	APMS6A × KMR3	E2A	114	111.63	9	23.76	3.23	194	89.57	16.31	18.32	5807.24
G3	APMS6A × KMR3	E2B	107	116.81	7	20.74	2.70	175	87.25	14.88	16.27	5104.98
G3	APMS6A × KMR3	E2C	116	112.50	7	21.43	2.87	188	87.37	14.98	12.17	3780.77
G21	IR58025A × KMR3	E1A	117	92.28	11	23.13	2.93	190	89.85	19.93	21.20	6755.06
G21	IR58025A × KMR3	E1B	110	92.41	10	22.16	2.59	188	88.42	20.22	20.27	6397.31
G21	IR58025A × KMR3	E1C	118	89.18	10	23.09	2.70	193	90.81	19.81	15.80	4979.31
G21	IR58025A × KMR3	E2A	115	90.24	12	23.77	2.90	200	89.51	19.89	20.63	6569.45
G21	IR58025A × KMR3	E2B	108	95.83	9	20.76	2.36	181	87.19	18.46	18.58	5867.19
G21	IR58025A × KMR3	E2C	116	92.29	9	21.45	2.53	194	87.32	18.56	14.48	4542.98
G41	IR79156A × RPHR1005	E1A	111	81.73	11	21.72	2.94	148	88.38	20.00	27.77	8925.82
G41	IR79156A × RPHR1005	E1B	104	81.72	11	20.75	2.60	145	86.96	20.29	26.85	8568.07
G41	IR79156A × RPHR1005	E1C	112	78.01	11	21.67	2.71	150	89.34	19.88	22.37	7150.07
G41	IR79156A × RPHR1005	E2A	109	79.69	12	22.36	2.90	158	88.05	19.96	27.20	8740.21
G41	IR79156A × RPHR1005	E2B	103	85.14	10	19.34	2.37	139	85.72	18.53	25.16	8037.95
G41	IR79156A × RPHR1005	E2C	111	81.25	10	20.04	2.54	152	85.85	18.63	21.05	6713.74
G57	IR68897A × KMR3	E1A	102	94.65	10	22.34	3.27	160	88.54	21.65	19.27	6120.82
G57	IR68897A × KMR3	E1B	95	94.58	9	21.37	2.93	158	87.11	21.94	18.35	5763.07
G57	IR68897A × KMR3	E1C	103	90.89	9	22.29	3.04	163	89.50	21.52	13.87	4345.07
G57	IR68897A × KMR3	E2A	100	92.75	11	22.98	3.23	170	88.20	21.61	18.71	5935.21
G57	IR68897A × KMR3	E2B	93	98.00	9	19.96	2.70	151	85.87	20.18	16.66	5232.95
G57	IR68897A × KMR3	E2C	102	94.61	8	20.66	2.87	164	86.00	20.28	12.55	3908.74
G74	IR58025B	E1A	116	106.39	11	21.97	2.96	155	89.13	19.31	26.01	8344.01
G74	IR58025B	E1B	109	106.42	10	21.00	2.62	152	87.70	19.60	25.08	7986.26

G74	IR58025B	E1C	117	102.49	11	21.92	2.73	157	90.09	19.19	20.61	6568.26
G74	IR58025B	E2A	114	104.35	12	22.61	2.92	165	88.79	19.27	25.44	8158.40
G74	IR58025B	E2B	107	109.88	10	19.59	2.39	146	86.47	17.84	23.40	7456.14
G74	IR58025B	E2C	116	105.60	10	20.29	2.56	159	86.59	17.94	19.29	6131.93
G83	IR40750R	E1A	121	99.19	10	22.08	2.94	182	86.14	17.39	17.43	5511.42
G83	IR40750R	E1B	113	99.16	9	21.11	2.60	180	84.71	17.68	16.50	5153.67
G83	IR40750R	E1C	121	95.37	10	22.04	2.71	185	87.10	17.26	12.03	3735.67
G83	IR40750R	E2A	118	97.44	11	22.72	2.90	193	85.80	17.35	16.86	5325.81
G83	IR40750R	E2B	112	102.58	9	19.71	2.37	173	83.48	15.92	14.81	4623.55
G83	IR40750R	E2C	120	98.65	8	20.40	2.54	186	83.60	16.02	10.71	3299.34
G85	C20R	E1A	119	99.92	11	23.56	3.22	145	86.48	19.24	19.34	6141.91
G85	C20R	E1B	112	100.00	11	22.59	2.87	143	85.05	19.53	18.41	5784.15
G85	C20R	E1C	119	96.19	11	23.51	2.99	148	87.44	19.12	13.94	4366.15
G85	C20R	E2A	116	97.88	12	24.20	3.18	155	86.14	19.20	18.77	5956.29
G85	C20R	E2B	110	103.42	10	21.18	2.64	136	83.82	17.77	16.72	5254.03
G85	C20R	E2C	118	99.48	10	21.88	2.82	149	83.94	17.87	12.62	3929.82
G98	KRH2	E1A	113	90.71	12	21.94	3.27	112	81.70	19.41	23.33	7460.81
G98	KRH2	E1B	106	90.90	11	20.97	2.93	110	80.27	19.70	22.41	7103.05
G98	KRH2	E1C	114	87.11	11	21.90	3.04	115	82.66	19.29	17.93	5685.05
G98	KRH2	E2A	111	88.67	12	22.58	3.23	123	81.36	19.37	22.77	7275.19
G98	KRH2	E2B	104	94.32	11	19.57	2.70	103	79.04	17.94	20.72	6572.93
G98	KRH2	E2C	112	90.37	10	20.26	2.87	117	79.16	18.04	16.61	5248.72
G100	IR64	E1A	113	94.76	11	22.55	2.25	106	87.74	20.73	17.32	5475.76
G100	IR64	E1B	106	94.44	10	21.58	1.91	104	86.31	21.02	16.39	5118.01
G100	IR64	E1C	113	91.34	11	22.50	2.02	109	88.70	20.61	11.92	3700.01
G100	IR64	E2A	110	92.72	12	23.19	2.22	117	87.40	20.69	16.75	5290.15
G100	IR64	E2B	104	97.86	10	20.18	1.68	97	85.08	19.26	14.70	4587.89
G100	IR64	E2C	112	94.45	9	20.87	1.85	110	85.21	19.36	10.60	3263.68
G102	Nagina22 (N22)	E1A	117	91.87	13	21.41	2.40	131	82.02	18.92	23.42	7487.76
G102	Nagina22 (N22)	E1B	110	92.12	12	20.44	2.05	129	80.59	19.21	22.49	7130.00

G102	Nagina22 (N22)	E1C	118	88.28	12	21.36	2.17	134	82.98	18.80	18.01	5712.00
G102	Nagina22 (N22)	E2A	115	89.97	14	22.05	2.36	141	81.68	18.88	22.85	7302.14
G102	Nagina22 (N22)	E2B	108	95.54	12	19.04	1.82	122	79.36	17.45	20.80	6599.88
G102	Nagina22 (N22)	E2C	116	91.54	11	19.73	2.00	135	79.48	17.55	16.70	5275.67

Supplementary Table 2. The mean data of all Parental lines Hybrids across the environments in the current study.

Code	Designation	ENV	DFF	PH	NRT	PL	PW	GP	SF	TW	SPY	YIELD
G1	APMS6A × BCW56	E1A	116	93.15	10	25.20	3.15	123	90.14	19.56	22.66	7238.88
G1	APMS6A × BCW56	E1B	109	92.98	9	24.23	2.81	120	88.71	19.85	21.74	6881.12
G1	APMS6A × BCW56	E1C	117	88.92	9	25.15	2.92	125	91.10	19.43	17.26	5463.13
G1	APMS6A × BCW56	E2A	114	91.11	10	25.84	3.12	133	89.80	19.52	22.09	7053.27
G1	APMS6A × BCW56	E2B	107	96.40	8	22.82	2.58	114	87.47	18.09	20.05	6351.01
G1	APMS6A × BCW56	E2C	116	92.18	8	23.52	2.75	127	87.60	18.19	15.94	5026.79
G2	APMS6A × EPLT104	E1A	113	108.59	11	20.40	2.85	164	83.29	18.81	22.67	7240.35
G2	APMS6A × EPLT104	E1B	106	108.60	10	19.43	2.51	162	81.87	19.10	21.74	6882.59
G2	APMS6A × EPLT104	E1C	113	105.04	10	20.35	2.62	166	84.26	18.69	17.27	5464.60
G2	APMS6A × EPLT104	E2A	110	106.55	11	21.04	2.81	174	82.96	18.77	22.10	7054.73
G2	APMS6A × EPLT104	E2B	104	112.06	9	18.02	2.28	155	80.63	17.34	20.05	6352.47
G2	APMS6A × EPLT104	E2C	112	107.97	9	18.72	2.45	168	80.76	17.44	15.95	5028.26
G3	APMS6A × KMR3	E1A	116	113.53	8	23.12	3.27	184	89.91	16.35	18.89	5992.86
G3	APMS6A × KMR3	E1B	109	113.35	8	22.15	2.93	182	88.48	16.64	17.96	5635.10
G3	APMS6A × KMR3	E1C	117	109.57	8	23.07	3.04	186	90.87	16.22	13.48	4217.10
G3	APMS6A × KMR3	E2A	114	111.63	9	23.76	3.23	194	89.57	16.31	18.32	5807.24
G3	APMS6A × KMR3	E2B	107	116.81	7	20.74	2.70	175	87.25	14.88	16.27	5104.98
G3	APMS6A × KMR3	E2C	116	112.50	7	21.43	2.87	188	87.37	14.98	12.17	3780.77
G4	APMS6A × RPHR619-2	E1A	114	106.94	12	23.33	3.33	150	88.42	18.48	24.09	7709.13
G4	APMS6A × RPHR619-2	E1B	107	106.91	12	22.36	2.98	148	87.00	18.77	23.16	7351.37
G4	APMS6A × RPHR619-2	E1C	114	103.21	12	23.28	3.09	153	89.39	18.36	18.69	5933.38
G4	APMS6A × RPHR619-2	E2A	112	104.90	13	23.97	3.29	161	88.09	18.44	23.52	7523.52
G4	APMS6A × RPHR619-2	E2B	105	110.37	11	20.95	2.75	141	85.76	17.01	21.47	6821.26
G4	APMS6A × RPHR619-2	E2C	113	106.14	11	21.65	2.92	154	85.89	17.11	17.37	5497.04
G5	APMS6A × RPHR1005	E1A	113	90.74	12	23.76	3.03	142	80.28	18.40	23.87	7637.36
G5	APMS6A × RPHR1005	E1B	106	90.98	11	22.78	2.69	140	78.86	18.69	22.94	7279.60

G5	APMS6A × RPHR1005	E1C	114	87.71	11	23.71	2.80	145	81.24	18.28	18.47	5861.60
G5	APMS6A × RPHR1005	E2A	111	88.85	13	24.40	3.00	153	79.95	18.36	23.30	7451.74
G5	APMS6A × RPHR1005	E2B	104	94.40	11	21.38	2.46	133	77.62	16.93	21.25	6749.48
G5	APMS6A × RPHR1005	E2C	113	90.81	10	22.07	2.63	146	77.75	17.03	17.15	5425.27
G6	APMS6A × RPHR517	E1A	112	92.94	12	23.58	3.55	221	91.58	19.02	19.83	6303.33
G6	APMS6A × RPHR517	E1B	105	93.16	11	22.61	3.20	219	90.16	19.31	18.90	5945.57
G6	APMS6A × RPHR517	E1C	112	89.35	12	23.53	3.32	223	92.54	18.89	14.43	4527.58
G6	APMS6A × RPHR517	E2A	109	90.90	13	24.22	3.51	231	91.25	18.98	19.26	6117.72
G6	APMS6A × RPHR517	E2B	103	96.58	11	21.20	2.98	212	88.92	17.54	17.21	5415.46
G6	APMS6A × RPHR517	E2C	111	92.64	10	21.90	3.15	225	89.05	17.65	13.11	4091.24
G7	APMS6A × IR40750R	E1A	111	104.96	12	23.15	3.25	134	80.56	18.49	24.46	7830.41
G7	APMS6A × IR40750R	E1B	103	105.17	12	22.18	2.91	132	79.14	18.79	23.53	7472.65
G7	APMS6A × IR40750R	E1C	111	101.65	12	23.10	3.02	137	81.53	18.37	19.05	6054.65
G7	APMS6A × IR40750R	E2A	108	103.06	13	23.79	3.21	145	80.23	18.45	23.89	7644.79
G7	APMS6A × IR40750R	E2B	101	108.63	11	20.78	2.68	125	77.90	17.02	21.84	6942.53
G7	APMS6A × IR40750R	E2C	110	104.58	10	21.47	2.85	138	78.03	17.12	17.74	5618.32
G8	APMS6A × 50-10	E1A	107	92.28	10	22.23	2.93	132	86.43	20.70	25.57	8198.81
G8	APMS6A × 50-10	E1B	100	92.10	9	21.26	2.59	130	85.00	20.99	24.64	7841.06
G8	APMS6A × 50-10	E1C	107	89.16	10	22.18	2.70	135	87.39	20.58	20.17	6423.06
G8	APMS6A × 50-10	E2A	104	90.24	11	22.87	2.90	142	86.09	20.66	25.00	8013.20
G8	APMS6A × 50-10	E2B	98	95.52	9	19.86	2.36	123	83.76	19.23	22.96	7310.94
G8	APMS6A × 50-10	E2C	106	92.27	8	20.55	2.53	136	83.89	19.33	18.85	5986.73
G9	APMS6A × C20R	E1A	113	88.35	13	22.02	2.63	152	85.98	19.92	21.79	6952.70
G9	APMS6A × C20R	E1B	107	88.40	12	21.05	2.29	150	84.55	20.21	20.87	6594.94
G9	APMS6A × C20R	E1C	114	84.96	13	21.97	2.40	154	86.94	19.80	16.39	5176.95
G9	APMS6A × C20R	E2A	111	86.31	14	22.66	2.59	162	85.64	19.88	21.22	6767.08
G9	APMS6A × C20R	E2B	105	91.82	12	19.65	2.06	143	83.32	18.45	19.18	6064.82
G9	APMS6A × C20R	E2C	113	88.23	12	20.34	2.23	156	83.44	18.55	15.07	4740.61
G10	APMS6A × RPHR695-1	E1A	112	98.76	12	24.58	2.95	130	82.80	18.77	28.09	9031.79
G10	APMS6A × RPHR695-1	E1B	106	98.21	11	23.61	2.61	128	81.37	19.06	27.17	8674.03

G10	APMS6A × RPHR695-1	E1C	113	95.30	12	24.53	2.72	133	83.76	18.65	22.69	7256.04
G10	APMS6A × RPHR695-1	E2A	110	96.72	13	25.22	2.91	141	82.46	18.73	27.53	8846.17
G10	APMS6A × RPHR695-1	E2B	104	101.63	11	22.20	2.38	121	80.14	17.30	25.48	8143.91
G10	APMS6A × RPHR695-1	E2C	112	98.28	10	22.90	2.55	134	80.26	17.40	21.38	6819.70
G11	APMS6A × IR-66R	E1A	118	93.92	12	22.33	2.68	121	82.88	21.73	21.28	6783.85
G11	APMS6A × IR-66R	E1B	110	93.96	11	21.36	2.34	119	81.45	22.02	20.36	6426.09
G11	APMS6A × IR-66R	E1C	118	90.54	12	22.28	2.45	124	83.84	21.61	15.88	5008.10
G11	APMS6A × IR-66R	E2A	115	92.03	13	22.97	2.64	131	82.54	21.69	20.71	6598.23
G11	APMS6A × IR-66R	E2B	109	97.38	11	19.95	2.11	112	80.22	20.26	18.67	5895.97
G11	APMS6A × IR-66R	E2C	116	93.64	10	20.65	2.28	125	80.34	20.36	14.56	4571.76
G12	APMS6A × Akshayadhan	E1A	112	77.19	12	23.04	2.59	157	89.30	19.55	24.03	7690.06
G12	APMS6A × Akshayadhan	E1B	105	77.61	12	22.07	2.25	155	87.87	19.84	23.10	7332.31
G12	APMS6A × Akshayadhan	E1C	113	74.01	12	22.99	2.36	159	90.26	19.43	18.63	5914.31
G12	APMS6A × Akshayadhan	E2A	110	75.15	13	23.68	2.55	167	88.96	19.51	23.46	7504.45
G12	APMS6A × Akshayadhan	E2B	104	81.03	11	20.66	2.02	148	86.64	18.08	21.42	6802.19
G12	APMS6A × Akshayadhan	E2C	111	77.25	11	21.36	2.19	161	86.76	18.18	17.31	5477.98
G13	APMS6A × SG27-105	E1A	111	80.81	11	22.58	2.92	156	89.29	19.45	23.37	7471.35
G13	APMS6A × SG27-105	E1B	104	80.85	10	21.61	2.58	154	87.87	19.74	22.44	7113.59
G13	APMS6A × SG27-105	E1C	111	77.48	11	22.53	2.69	159	90.26	19.32	17.97	5695.60
G13	APMS6A × SG27-105	E2A	108	78.77	12	23.22	2.89	166	88.96	19.41	22.80	7285.73
G13	APMS6A × SG27-105	E2B	102	84.27	10	20.20	2.35	147	86.63	17.98	20.75	6583.47
G13	APMS6A × SG27-105	E2C	110	80.72	9	20.90	2.52	160	86.76	18.08	16.65	5259.26
G14	APMS6A × 363-5	E1A	109	95.90	13	21.54	2.63	148	86.74	17.15	22.37	7141.35
G14	APMS6A × 363-5	E1B	103	95.47	12	20.57	2.28	145	85.31	17.44	21.44	6783.59
G14	APMS6A × 363-5	E1C	110	92.40	13	21.49	2.39	150	87.70	17.02	16.97	5365.60
G14	APMS6A × 363-5	E2A	107	93.86	13	22.18	2.59	158	86.40	17.11	21.80	6955.73
G14	APMS6A × 363-5	E2B	101	98.89	12	19.16	2.05	139	84.08	15.68	19.75	6253.47
G14	APMS6A × 363-5	E2C	108	95.53	11	19.86	2.22	152	84.20	15.78	15.65	4929.26
G15	APMS6A × RPHR611-1	E1A	109	93.16	12	21.50	2.13	108	86.48	18.95	17.02	5375.85
G15	APMS6A × RPHR611-1	E1B	101	93.31	11	20.53	1.79	106	85.05	19.24	16.09	5018.09

G15	APMS6A × RPHR611-1	E1C	108	89.82	12	21.46	1.90	110	87.44	18.82	11.62	3600.10
G15	APMS6A × RPHR611-1	E2A	106	91.12	13	22.14	2.09	118	86.14	18.91	16.45	5190.23
G15	APMS6A × RPHR611-1	E2B	99	96.73	11	19.13	1.56	99	83.82	17.48	14.40	4487.97
G15	APMS6A × RPHR611-1	E2C	107	93.10	10	19.82	1.73	112	83.94	17.58	10.30	3163.76
G16	APMS6A × IBL57	E1A	114	98.38	11	22.86	2.98	151	88.12	19.21	18.53	5875.52
G16	APMS6A × IBL57	E1B	107	98.39	11	21.89	2.64	149	86.69	19.50	17.61	5517.77
G16	APMS6A × IBL57	E1C	115	95.16	11	22.81	2.75	154	89.08	19.09	13.13	4099.77
G16	APMS6A × IBL57	E2A	112	96.49	12	23.50	2.94	162	87.78	19.17	17.96	5689.91
G16	APMS6A × IBL57	E2B	105	101.81	10	20.48	2.41	142	85.45	17.74	15.92	4987.65
G16	APMS6A × IBL57	E2C	113	98.27	10	21.18	2.58	155	85.58	17.84	11.81	3663.44
G17	APMS6A × BK-49-180	E1A	105	97.01	13	22.51	2.44	129	90.18	19.89	16.69	5267.31
G17	APMS6A × BK-49-180	E1B	99	97.01	12	21.54	2.10	126	88.75	20.18	15.76	4909.56
G17	APMS6A × BK-49-180	E1C	106	93.70	13	22.46	2.21	131	91.14	19.76	11.29	3491.56
G17	APMS6A × BK-49-180	E2A	103	95.25	14	23.15	2.40	139	89.84	19.85	16.12	5081.70
G17	APMS6A × BK-49-180	E2B	97	100.43	12	20.14	1.87	120	87.52	18.42	14.07	4379.44
G17	APMS6A × BK-49-180	E2C	104	96.83	11	20.83	2.04	133	87.64	18.52	9.97	3055.23
G18	APMS6A × RPHR1096	E1A	115	103.03	11	23.80	3.22	130	80.08	17.44	16.28	5134.12
G18	APMS6A × RPHR1096	E1B	108	103.09	11	22.83	2.88	127	78.66	17.73	15.36	4776.37
G18	APMS6A × RPHR1096	E1C	115	99.52	11	23.75	2.99	132	81.04	17.32	10.88	3358.37
G18	APMS6A × RPHR1096	E2A	112	101.14	12	24.44	3.18	140	79.75	17.40	15.71	4948.51
G18	APMS6A × RPHR1096	E2B	106	106.53	10	21.42	2.65	121	77.42	15.97	13.67	4246.25
G18	APMS6A × RPHR1096	E2C	114	102.63	9	22.12	2.82	134	77.55	16.07	9.56	2922.04
G19	IR58025A × BCW56	E1A	107	91.15	13	23.83	2.72	155	89.46	19.00	21.98	7013.20
G19	IR58025A × BCW56	E1B	100	91.45	12	22.86	2.38	153	88.04	19.29	21.05	6655.44
G19	IR58025A × BCW56	E1C	107	88.13	12	23.78	2.49	158	90.42	18.87	16.58	5237.45
G19	IR58025A × BCW56	E2A	105	89.11	13	24.47	2.68	166	89.13	18.96	21.41	6827.58
G19	IR58025A × BCW56	E2B	98	94.87	11	21.45	2.15	146	86.80	17.53	19.36	6125.32
G19	IR58025A × BCW56	E2C	106	91.19	11	22.15	2.32	159	86.93	17.63	15.26	4801.11
G20	IR58025A × EPLT104	E1A	115	95.04	12	23.13	3.21	154	82.19	16.64	24.70	7910.61
G20	IR58025A × EPLT104	E1B	108	95.11	11	22.16	2.87	151	80.76	16.93	23.77	7552.86

G20	IR58025A × EPLT104	E1C	115	91.63	12	23.08	2.98	156	83.15	16.51	19.30	6134.86
G20	IR58025A × EPLT104	E2A	112	93.00	13	23.77	3.18	164	81.85	16.60	24.13	7725.00
G20	IR58025A × EPLT104	E2B	106	98.53	11	20.75	2.64	145	79.52	15.17	22.08	7022.74
G20	IR58025A × EPLT104	E2C	114	94.91	10	21.44	2.81	158	79.65	15.27	17.98	5698.53
G21	IR58025A × KMR3	E1A	117	92.28	11	23.13	2.93	190	89.85	19.93	21.20	6755.06
G21	IR58025A × KMR3	E1B	110	92.41	10	22.16	2.59	188	88.42	20.22	20.27	6397.31
G21	IR58025A × KMR3	E1C	118	89.18	10	23.09	2.70	193	90.81	19.81	15.80	4979.31
G21	IR58025A × KMR3	E2A	115	90.24	12	23.77	2.90	200	89.51	19.89	20.63	6569.45
G21	IR58025A × KMR3	E2B	108	95.83	9	20.76	2.36	181	87.19	18.46	18.58	5867.19
G21	IR58025A × KMR3	E2C	116	92.29	9	21.45	2.53	194	87.32	18.56	14.48	4542.98
G22	IR58025A × RPHR619-2	E1A	114	89.95	12	23.83	3.20	175	86.29	18.08	26.86	8520.16
G22	IR58025A × RPHR619-2	E1B	107	89.82	11	22.86	2.85	173	84.86	18.37	25.93	8162.41
G22	IR58025A × RPHR619-2	E1C	115	86.28	11	23.78	2.97	178	87.25	17.96	21.46	6744.41
G22	IR58025A × RPHR619-2	E2A	111	87.91	12	24.47	3.16	186	85.95	18.04	26.29	8334.55
G22	IR58025A × RPHR619-2	E2B	105	93.24	11	21.45	2.63	166	83.62	16.61	24.25	7632.29
G22	IR58025A × RPHR619-2	E2C	113	89.54	10	22.15	2.80	179	83.75	16.71	20.14	6308.08
G23	IR58025A × RPHR1005	E1A	98	105.40	8	23.44	3.09	159	87.61	22.36	23.27	7329.21
G23	IR58025A × RPHR1005	E1B	91	105.30	7	22.47	2.74	157	86.18	22.65	22.34	6971.46
G23	IR58025A × RPHR1005	E1C	98	101.92	8	23.39	2.86	162	88.57	22.24	17.87	5553.46
G23	IR58025A × RPHR1005	E2A	96	103.64	9	24.08	3.05	170	87.27	22.32	22.70	7143.60
G23	IR58025A × RPHR1005	E2B	89	108.72	7	21.06	2.51	150	84.95	20.89	20.65	6441.34
G23	IR58025A × RPHR1005	E2C	97	105.10	6	21.76	2.69	163	85.08	20.99	16.55	5117.13
G24	IR58025A × RPHR517	E1A	117	113.75	10	23.54	3.35	154	90.61	18.97	20.60	6559.72
G24	IR58025A × RPHR517	E1B	110	113.98	10	22.57	3.01	152	89.19	19.26	19.68	6201.97
G24	IR58025A × RPHR517	E1C	118	110.28	10	23.49	3.12	157	91.57	18.85	15.20	4783.97
G24	IR58025A × RPHR517	E2A	115	111.99	11	24.18	3.31	165	90.28	18.93	20.04	6374.11
G24	IR58025A × RPHR517	E2B	108	117.44	9	21.16	2.78	145	87.95	17.50	17.99	5671.85
G24	IR58025A × RPHR517	E2C	116	113.21	9	21.86	2.95	159	88.08	17.60	13.88	4347.64
G25	IR58025A × IR40750R	E1A	109	106.87	11	23.46	2.92	160	81.11	19.16	21.06	6709.60
G25	IR58025A × IR40750R	E1B	102	106.79	10	22.49	2.58	158	79.68	19.45	20.13	6351.84

G25	IR58025A × IR40750R	E1C	109	103.24	10	23.41	2.69	163	82.07	19.03	15.66	4933.85
G25	IR58025A × IR40750R	E2A	106	104.83	12	24.10	2.88	171	80.77	19.12	20.49	6523.98
G25	IR58025A × IR40750R	E2B	100	110.25	10	21.08	2.35	151	78.45	17.68	18.44	5821.72
G25	IR58025A × IR40750R	E2C	108	106.17	9	21.78	2.52	165	78.57	17.79	14.34	4497.51
G26	IR58025A × 50-10	E1A	114	89.98	11	24.05	3.44	150	86.47	19.32	26.60	7802.85
G26	IR58025A × 50-10	E1B	107	89.97	11	23.08	3.10	147	85.04	19.61	25.68	7445.09
G26	IR58025A × 50-10	E1C	114	86.03	11	24.00	3.21	152	87.43	19.20	21.20	6027.10
G26	IR58025A × 50-10	E2A	111	88.22	12	24.69	3.41	160	86.13	19.28	26.03	7617.23
G26	IR58025A × 50-10	E2B	105	93.39	10	21.67	2.87	141	83.81	17.85	23.99	6914.97
G26	IR58025A × 50-10	E2C	113	89.30	10	22.37	3.04	154	83.93	17.95	19.88	5590.76
G27	IR58025A × C20R	E1A	116	92.50	12	23.84	3.37	155	89.75	19.56	24.15	7730.95
G27	IR58025A × C20R	E1B	109	92.62	11	22.87	3.02	153	88.32	19.86	23.23	7373.19
G27	IR58025A × C20R	E1C	117	89.07	12	23.79	3.14	158	90.71	19.44	18.75	5955.20
G27	IR58025A × C20R	E2A	113	90.61	13	24.48	3.33	166	89.41	19.52	23.58	7545.33
G27	IR58025A × C20R	E2B	107	95.96	11	21.46	2.79	146	87.09	18.09	21.54	6843.07
G27	IR58025A × C20R	E2C	115	92.35	10	22.16	2.97	159	87.21	18.19	17.43	5518.86
G28	IR58025A × RPHR695-1	E1A	117	92.83	10	23.13	3.18	174	88.34	20.65	25.91	8310.83
G28	IR58025A × RPHR695-1	E1B	110	92.46	9	22.16	2.83	172	86.91	20.94	24.98	7953.07
G28	IR58025A × RPHR695-1	E1C	117	88.96	10	23.09	2.95	177	89.30	20.52	20.51	6535.08
G28	IR58025A × RPHR695-1	E2A	114	90.94	10	23.77	3.14	184	88.00	20.61	25.34	8125.22
G28	IR58025A × RPHR695-1	E2B	108	95.88	9	20.76	2.60	165	85.68	19.18	23.30	7422.96
G28	IR58025A × RPHR695-1	E2C	116	92.23	8	21.45	2.78	178	85.80	19.28	19.19	6098.74
G29	IR58025A × IR-66R	E1A	111	96.29	13	24.86	2.17	115	82.56	16.72	18.72	5937.21
G29	IR58025A × IR-66R	E1B	104	96.36	13	23.89	1.83	112	81.13	17.01	17.79	5579.46
G29	IR58025A × IR-66R	E1C	111	92.72	13	24.81	1.94	117	83.52	16.59	13.32	4161.46
G29	IR58025A × IR-66R	E2A	108	94.25	14	25.50	2.13	125	82.22	16.68	18.15	5751.60
G29	IR58025A × IR-66R	E2B	102	99.78	12	22.48	1.60	106	79.90	15.25	16.10	5049.34
G29	IR58025A × IR-66R	E2C	110	95.83	12	23.18	1.77	119	80.02	15.35	12.00	3725.13
G30	IR58025A × Akshayadhan	E1A	112	97.44	10	23.39	2.89	160	86.10	17.97	20.64	6572.37
G30	IR58025A × Akshayadhan	E1B	105	97.89	10	22.41	2.55	158	84.67	18.26	19.72	6214.62

G30	IR58025A × Akshayadhan	E1C	112	93.84	10	23.34	2.66	163	87.06	17.84	15.24	4796.62
G30	IR58025A × Akshayadhan	E2A	109	95.40	11	24.03	2.85	171	85.76	17.93	20.07	6386.76
G30	IR58025A × Akshayadhan	E2B	103	101.33	9	21.01	2.32	151	83.44	16.50	18.03	5684.50
G30	IR58025A × Akshayadhan	E2C	111	97.13	9	21.70	2.49	165	83.56	16.60	13.92	4360.29
G31	IR58025A × SG27-105	E1A	118	97.57	11	23.27	3.09	162	90.87	20.32	22.23	7097.71
G31	IR58025A × SG27-105	E1B	111	97.59	11	22.30	2.75	159	89.45	20.61	21.31	6739.96
G31	IR58025A × SG27-105	E1C	119	93.84	11	23.23	2.86	164	91.83	20.20	16.83	5321.96
G31	IR58025A × SG27-105	E2A	116	95.67	12	23.91	3.05	172	90.54	20.28	21.66	6912.10
G31	IR58025A × SG27-105	E2B	109	101.01	10	20.90	2.52	153	88.21	18.85	19.62	6209.84
G31	IR58025A × SG27-105	E2C	118	97.12	10	21.59	2.69	166	88.34	18.95	15.51	4885.63
G32	IR58025A × 363-5	E1A	111	79.51	10	22.52	2.33	136	81.96	19.86	22.05	7036.76
G32	IR58025A × 363-5	E1B	103	79.57	9	21.55	1.99	134	80.53	20.15	21.13	6679.00
G32	IR58025A × 363-5	E1C	111	75.91	10	22.47	2.10	138	82.92	19.73	16.65	5261.00
G32	IR58025A × 363-5	E2A	108	77.47	11	23.16	2.29	146	81.62	19.82	21.48	6851.14
G32	IR58025A × 363-5	E2B	102	82.99	9	20.14	1.76	127	79.30	18.38	19.44	6148.88
G32	IR58025A × 363-5	E2C	110	79.15	8	20.84	1.93	140	79.42	18.49	15.33	4824.67
G33	IR58025A × RPHR611-1	E1A	117	94.00	11	23.20	3.08	150	92.85	20.20	24.49	7842.69
G33	IR58025A × RPHR611-1	E1B	110	94.55	10	22.23	2.74	148	91.42	20.49	23.57	7484.93
G33	IR58025A × RPHR611-1	E1C	118	90.88	11	23.15	2.85	153	93.81	20.08	19.09	6066.94
G33	IR58025A × RPHR611-1	E2A	115	92.11	12	23.84	3.05	160	92.51	20.16	23.92	7657.07
G33	IR58025A × RPHR611-1	E2B	108	97.97	10	20.82	2.51	141	90.19	18.73	21.88	6954.81
G33	IR58025A × RPHR611-1	E2C	116	93.99	10	21.52	2.68	154	90.31	18.83	17.77	5630.60
G34	IR58025A × IBL57	E1A	105	95.38	13	24.45	3.37	171	86.59	19.79	24.28	7773.39
G34	IR58025A × IBL57	E1B	98	95.76	12	23.48	3.03	169	85.16	20.08	23.36	7415.63
G34	IR58025A × IBL57	E1C	105	91.71	12	24.40	3.14	174	87.55	19.66	18.88	5997.64
G34	IR58025A × IBL57	E2A	102	93.34	13	25.09	3.33	182	86.25	19.75	23.71	7587.77
G34	IR58025A × IBL57	E2B	96	99.18	12	22.07	2.80	162	83.92	18.32	21.67	6885.51
G34	IR58025A × IBL57	E2C	104	95.21	11	22.77	2.97	176	84.05	18.42	17.56	5561.30
G35	IR58025A × BK-49-180	E1A	120	101.03	9	23.23	3.14	180	90.82	16.65	24.24	7437.94
G35	IR58025A × BK-49-180	E1B	113	100.95	9	22.26	2.79	177	89.39	16.94	23.32	7080.18

G35	IR58025A × BK-49-180	E1C	120	97.45	9	23.18	2.91	182	91.78	16.52	18.84	5662.19
G35	IR58025A × BK-49-180	E2A	117	99.13	10	23.87	3.10	190	90.48	16.61	23.68	7252.32
G35	IR58025A × BK-49-180	E2B	111	104.37	8	20.86	2.56	171	88.16	15.17	21.63	6550.06
G35	IR58025A × BK-49-180	E2C	119	100.56	8	21.55	2.74	184	88.28	15.28	17.52	5225.85
G36	IR58025A × RPHR1096	E1A	117	105.47	9	23.50	3.01	173	86.81	20.64	21.37	6811.07
G36	IR58025A × RPHR1096	E1B	110	105.52	9	22.53	2.67	171	85.38	20.93	20.44	6453.32
G36	IR58025A × RPHR1096	E1C	117	101.73	9	23.45	2.78	176	87.77	20.52	15.96	5035.32
G36	IR58025A × RPHR1096	E2A	114	103.43	10	24.14	2.97	184	86.47	20.60	20.80	6625.46
G36	IR58025A × RPHR1096	E2B	108	108.98	9	21.12	2.44	164	84.15	19.17	18.75	5923.20
G36	IR58025A × RPHR1096	E2C	116	104.66	8	21.82	2.61	177	84.28	19.27	14.65	4598.99
G37	IR79156A × BCW56	E1A	109	96.84	10	23.72	3.00	129	86.29	18.86	20.94	6670.18
G37	IR79156A × BCW56	E1B	102	96.77	10	22.75	2.66	126	84.87	19.15	20.01	6312.42
G37	IR79156A × BCW56	E1C	109	93.01	10	23.67	2.77	131	87.25	18.74	15.54	4894.43
G37	IR79156A × BCW56	E2A	106	94.80	11	24.36	2.96	139	85.96	18.82	20.37	6484.57
G37	IR79156A × BCW56	E2B	100	100.19	9	21.34	2.43	120	83.63	17.39	18.33	5782.31
G37	IR79156A × BCW56	E2C	108	96.29	9	22.04	2.60	133	83.76	17.49	14.22	4458.09
G38	IR79156A × EPLT104	E1A	115	98.17	12	22.14	3.24	150	88.07	19.07	21.66	6909.63
G38	IR79156A × EPLT104	E1B	108	98.34	12	21.17	2.90	147	86.65	19.36	20.74	6551.88
G38	IR79156A × EPLT104	E1C	116	94.67	12	22.09	3.01	152	89.03	18.95	16.26	5133.88
G38	IR79156A × EPLT104	E2A	113	96.27	13	22.78	3.21	160	87.74	19.03	21.09	6724.02
G38	IR79156A × EPLT104	E2B	106	101.76	11	19.77	2.67	141	85.41	17.60	19.05	6021.76
G38	IR79156A × EPLT104	E2C	114	97.95	11	20.46	2.84	154	85.54	17.70	14.94	4697.55
G39	IR79156A × KMR3	E1A	106	95.10	10	23.59	3.69	148	90.15	20.57	25.50	8176.63
G39	IR79156A × KMR3	E1B	99	95.18	9	22.62	3.34	146	88.73	20.87	24.58	7818.87
G39	IR79156A × KMR3	E1C	107	91.50	9	23.54	3.46	151	91.11	20.45	20.10	6400.88
G39	IR79156A × KMR3	E2A	104	93.06	10	24.23	3.65	158	89.82	20.53	24.94	7991.02
G39	IR79156A × KMR3	E2B	97	98.60	8	21.21	3.11	139	87.49	19.10	22.89	7288.76
G39	IR79156A × KMR3	E2C	105	94.79	8	21.91	3.29	152	87.62	19.20	18.79	5964.54
G40	IR79156A × RPHR619-2	E1A	117	96.32	12	23.77	2.71	139	89.70	18.73	21.94	7002.29
G40	IR79156A × RPHR619-2	E1B	110	96.31	12	22.80	2.36	137	88.27	19.02	21.02	6644.53

G40	IR79156A × RPHR619-2	E1C	118	92.37	12	23.72	2.48	141	90.66	18.60	16.54	5226.54
G40	IR79156A × RPHR619-2	E2A	115	94.42	13	24.41	2.67	149	89.36	18.69	21.38	6816.67
G40	IR79156A × RPHR619-2	E2B	108	99.73	11	21.39	2.14	130	87.03	17.26	19.33	6114.41
G40	IR79156A × RPHR619-2	E2C	117	95.66	11	22.09	2.31	143	87.16	17.36	15.22	4790.20
G41	IR79156A × RPHR1005	E1A	111	81.73	11	21.72	2.94	148	88.38	20.00	27.77	8925.82
G41	IR79156A × RPHR1005	E1B	104	81.72	11	20.75	2.60	145	86.96	20.29	26.85	8568.07
G41	IR79156A × RPHR1005	E1C	112	78.01	11	21.67	2.71	150	89.34	19.88	22.37	7150.07
G41	IR79156A × RPHR1005	E2A	109	79.69	12	22.36	2.90	158	88.05	19.96	27.20	8740.21
G41	IR79156A × RPHR1005	E2B	103	85.14	10	19.34	2.37	139	85.72	18.53	25.16	8037.95
G41	IR79156A × RPHR1005	E2C	111	81.25	10	20.04	2.54	152	85.85	18.63	21.05	6713.74
G42	IR79156A × RPHR517	E1A	110	83.26	12	23.83	2.93	165	88.20	19.02	21.78	6946.74
G42	IR79156A × RPHR517	E1B	103	83.04	12	22.86	2.59	163	86.78	19.31	20.85	6588.98
G42	IR79156A × RPHR517	E1C	111	79.34	12	23.78	2.70	168	89.16	18.90	16.37	5170.99
G42	IR79156A × RPHR517	E2A	108	81.22	13	24.47	2.90	176	87.87	18.98	21.21	6761.12
G42	IR79156A × RPHR517	E2B	102	86.46	11	21.46	2.36	156	85.54	17.55	19.16	6058.86
G42	IR79156A × RPHR517	E2C	110	82.58	11	22.15	2.53	169	85.67	17.65	15.06	4734.65
G43	IR79156A × IR40750R	E1A	106	97.62	11	24.53	2.90	148	90.96	20.16	29.30	9430.26
G43	IR79156A × IR40750R	E1B	98	97.77	11	23.56	2.56	146	89.53	20.46	28.38	9072.51
G43	IR79156A × IR40750R	E1C	106	94.31	11	24.48	2.67	151	91.92	20.04	23.90	7654.51
G43	IR79156A × IR40750R	E2A	103	95.58	12	25.17	2.87	159	90.62	20.12	28.73	9244.65
G43	IR79156A × IR40750R	E2B	96	101.19	10	22.15	2.33	139	88.30	18.69	26.69	8542.39
G43	IR79156A × IR40750R	E2C	104	97.42	10	22.85	2.50	152	88.43	18.79	22.58	7218.18
G44	IR79156A × 50-10	E1A	114	93.82	10	22.91	3.21	158	89.46	18.82	24.65	7894.48
G44	IR79156A × 50-10	E1B	107	94.00	10	21.94	2.87	156	88.04	19.11	23.72	7536.72
G44	IR79156A × 50-10	E1C	114	90.05	10	22.86	2.98	161	90.42	18.70	19.25	6118.73
G44	IR79156A × 50-10	E2A	112	91.92	11	23.55	3.17	169	89.13	18.78	24.08	7708.87
G44	IR79156A × 50-10	E2B	105	97.42	9	20.54	2.64	149	86.80	17.35	22.03	7006.61
G44	IR79156A × 50-10	E2C	113	93.44	9	21.23	2.81	162	86.93	17.45	17.93	5682.39
G45	IR79156A × C20R	E1A	118	101.51	11	22.69	2.93	173	87.01	19.04	28.81	9269.21
G45	IR79156A × C20R	E1B	111	101.52	11	21.72	2.59	171	85.58	19.33	27.89	8911.45

G45	IR79156A × C20R	E1C	118	97.90	11	22.64	2.70	176	87.97	18.92	23.41	7493.45
G45	IR79156A × C20R	E2A	115	99.61	12	23.33	2.89	183	86.67	19.00	28.25	9083.59
G45	IR79156A × C20R	E2B	109	104.94	10	20.31	2.36	164	84.34	17.57	26.20	8381.33
G45	IR79156A × C20R	E2C	117	101.01	10	21.01	2.53	177	84.47	17.67	22.10	7057.12
G46	IR79156A × RPHR695-1	E1A	108	98.92	11	23.43	2.70	135	89.26	20.11	19.57	6219.64
G46	IR79156A × RPHR695-1	E1B	101	98.69	10	22.46	2.36	133	87.84	20.40	18.65	5861.88
G46	IR79156A × RPHR695-1	E1C	108	95.17	11	23.38	2.47	137	90.23	19.98	14.17	4443.89
G46	IR79156A × RPHR695-1	E2A	105	96.88	12	24.07	2.67	145	88.93	20.07	19.01	6034.02
G46	IR79156A × RPHR695-1	E2B	99	102.11	10	21.05	2.13	126	86.60	18.64	16.96	5331.76
G46	IR79156A × RPHR695-1	E2C	107	98.45	10	21.74	2.30	139	86.73	18.74	12.86	4007.55
G47	IR79156A × IR-66R	E1A	120	103.64	12	24.44	3.29	153	87.72	18.62	24.44	7826.65
G47	IR79156A × IR-66R	E1B	113	103.52	11	23.47	2.94	150	86.29	18.91	23.52	7468.89
G47	IR79156A × IR-66R	E1C	120	99.90	12	24.39	3.05	155	88.68	18.50	19.04	6050.90
G47	IR79156A × IR-66R	E2A	117	101.74	13	25.08	3.25	163	87.38	18.58	23.87	7641.03
G47	IR79156A × IR-66R	E2B	111	106.96	11	22.06	2.71	144	85.06	17.15	21.83	6938.77
G47	IR79156A × IR-66R	E2C	119	103.18	10	22.76	2.88	157	85.18	17.25	17.72	5614.56
G48	IR79156A × Akshayadhan	E1A	112	90.37	11	21.81	2.36	136	86.29	18.86	18.65	5915.76
G48	IR79156A × Akshayadhan	E1B	105	90.62	11	20.84	2.02	134	84.86	19.15	17.73	5558.01
G48	IR79156A × Akshayadhan	E1C	112	86.48	11	21.77	2.13	139	87.25	18.74	13.25	4140.01
G48	IR79156A × Akshayadhan	E2A	109	88.33	12	22.45	2.33	146	85.95	18.82	18.08	5730.15
G48	IR79156A × Akshayadhan	E2B	103	94.05	10	19.44	1.79	127	83.63	17.39	16.04	5027.89
G48	IR79156A × Akshayadhan	E2C	111	89.72	10	20.13	1.96	140	83.76	17.49	11.93	3703.68
G49	IR79156A × SG27-105	E1A	115	99.14	12	20.85	3.21	161	88.42	15.43	20.98	6683.01
G49	IR79156A × SG27-105	E1B	108	99.02	11	19.88	2.87	158	86.99	15.72	20.05	6325.26
G49	IR79156A × SG27-105	E1C	116	95.80	11	20.80	2.98	163	89.38	15.31	15.58	4907.26
G49	IR79156A × SG27-105	E2A	113	97.10	12	21.49	3.17	171	88.08	15.39	20.41	6497.40
G49	IR79156A × SG27-105	E2B	107	102.44	11	18.48	2.64	152	85.76	13.96	18.36	5795.14
G49	IR79156A × SG27-105	E2C	115	98.74	10	19.17	2.81	165	85.88	14.06	14.26	4470.93
G50	IR79156A × 363-5	E1A	116	96.12	12	23.06	2.79	109	90.24	18.24	18.86	5983.32
G50	IR79156A × 363-5	E1B	109	96.03	11	22.09	2.44	107	88.82	18.53	17.93	5625.57

G50	IR79156A × 363-5	E1C	116	92.19	12	23.01	2.56	111	91.20	18.11	13.46	4207.57
G50	IR79156A × 363-5	E2A	113	94.22	13	23.70	2.75	119	89.91	18.20	18.29	5797.71
G50	IR79156A × 363-5	E2B	107	99.45	11	20.69	2.21	100	87.58	16.77	16.24	5095.45
G50	IR79156A × 363-5	E2C	115	95.48	10	21.38	2.39	113	87.71	16.87	12.14	3771.24
G51	IR79156A × RPHR611-1	E1A	110	83.88	11	22.87	3.19	165	88.95	16.11	30.44	8607.07
G51	IR79156A × RPHR611-1	E1B	103	83.88	10	21.89	2.84	163	87.52	16.41	29.51	8249.32
G51	IR79156A × RPHR611-1	E1C	111	80.09	11	22.82	2.96	168	89.91	15.99	25.04	6831.32
G51	IR79156A × RPHR611-1	E2A	107	81.84	12	23.51	3.15	175	88.61	16.07	29.87	8421.46
G51	IR79156A × RPHR611-1	E2B	101	87.18	10	20.49	2.62	156	86.29	14.64	27.82	7719.20
G51	IR79156A × RPHR611-1	E2C	109	83.33	9	21.18	2.79	169	86.41	14.74	23.72	6394.99
G52	IR79156A × IBL57	E1A	101	87.38	11	22.87	2.42	153	88.48	18.88	18.12	5739.76
G52	IR79156A × IBL57	E1B	94	87.20	11	21.90	2.08	150	87.06	19.17	17.19	5382.01
G52	IR79156A × IBL57	E1C	101	83.80	11	22.82	2.19	155	89.44	18.76	12.72	3964.01
G52	IR79156A × IBL57	E2A	98	85.50	12	23.51	2.38	163	88.15	18.84	17.55	5554.15
G52	IR79156A × IBL57	E2B	92	90.62	10	20.49	1.85	143	85.82	17.41	15.50	4851.89
G52	IR79156A × IBL57	E2C	100	87.07	10	21.18	2.02	157	85.95	17.51	11.40	3527.68
G53	IR79156A × BK-49-180	E1A	103	85.43	12	24.68	3.05	144	86.88	20.76	21.11	6725.73
G53	IR79156A × BK-49-180	E1B	96	85.49	11	23.71	2.71	142	85.46	21.05	20.18	6367.97
G53	IR79156A × BK-49-180	E1C	104	81.44	12	24.63	2.82	147	87.85	20.64	15.71	4949.98
G53	IR79156A × BK-49-180	E2A	101	83.53	13	25.32	3.02	154	86.55	20.72	20.54	6540.12
G53	IR79156A × BK-49-180	E2B	95	88.92	11	22.30	2.48	135	84.22	19.29	18.49	5837.86
G53	IR79156A × BK-49-180	E2C	103	84.68	10	23.00	2.65	148	84.35	19.39	14.39	4513.64
G54	IR79156A × RPHR1096	E1A	108	93.67	11	23.03	3.14	135	88.11	16.54	20.49	6522.87
G54	IR79156A × RPHR1096	E1B	101	93.62	10	22.06	2.80	133	86.68	16.83	19.57	6165.12
G54	IR79156A × RPHR1096	E1C	108	89.89	11	22.98	2.91	138	89.07	16.42	15.09	4747.12
G54	IR79156A × RPHR1096	E2A	106	91.77	12	23.67	3.11	146	87.77	16.50	19.92	6337.26
G54	IR79156A × RPHR1096	E2B	99	97.04	10	20.66	2.57	126	85.45	15.07	17.88	5635.00
G54	IR79156A × RPHR1096	E2C	107	93.17	9	21.35	2.74	140	85.57	15.17	13.77	4310.79
G55	IR68897A × BCW56	E1A	103	97.26	12	23.63	2.64	130	84.21	18.09	18.74	5944.36
G55	IR68897A × BCW56	E1B	97	97.19	12	22.66	2.30	127	82.79	18.38	17.81	5586.61

G55	IR68897A × BCW56	E1C	104	93.39	12	23.58	2.41	132	85.17	17.97	13.34	4168.61
G55	IR68897A × BCW56	E2A	101	95.36	13	24.27	2.60	140	83.88	18.05	18.17	5758.75
G55	IR68897A × BCW56	E2B	95	100.61	11	21.25	2.07	120	81.55	16.62	16.12	5056.49
G55	IR68897A × BCW56	E2C	103	96.67	11	21.95	2.24	134	81.68	16.72	12.02	3732.28
G56	IR68897A × EPLT104	E1A	105	90.88	13	23.00	2.89	142	87.08	18.81	20.81	6628.20
G56	IR68897A × EPLT104	E1B	98	90.92	12	22.03	2.55	140	85.65	19.10	19.88	6270.44
G56	IR68897A × EPLT104	E1C	105	87.25	12	22.95	2.66	145	88.04	18.68	15.41	4852.45
G56	IR68897A × EPLT104	E2A	102	88.84	13	23.64	2.86	153	86.74	18.77	20.24	6442.58
G56	IR68897A × EPLT104	E2B	96	94.34	11	20.63	2.32	133	84.42	17.34	18.20	5740.32
G56	IR68897A × EPLT104	E2C	104	90.51	11	21.32	2.49	146	84.55	17.44	14.09	4416.11
G57	IR68897A × KMR3	E1A	102	94.65	10	22.34	3.27	160	88.54	21.65	19.27	6120.82
G57	IR68897A × KMR3	E1B	95	94.58	9	21.37	2.93	158	87.11	21.94	18.35	5763.07
G57	IR68897A × KMR3	E1C	103	90.89	9	22.29	3.04	163	89.50	21.52	13.87	4345.07
G57	IR68897A × KMR3	E2A	100	92.75	11	22.98	3.23	170	88.20	21.61	18.71	5935.21
G57	IR68897A × KMR3	E2B	93	98.00	9	19.96	2.70	151	85.87	20.18	16.66	5232.95
G57	IR68897A × KMR3	E2C	102	94.61	8	20.66	2.87	164	86.00	20.28	12.55	3908.74
G58	IR68897A × RPHR619-2	E1A	106	86.79	14	22.28	3.28	167	90.45	17.59	19.64	6242.65
G58	IR68897A × RPHR619-2	E1B	99	86.67	13	21.31	2.94	165	89.02	17.88	18.72	5884.89
G58	IR68897A × RPHR619-2	E1C	107	83.10	14	22.23	3.05	170	91.41	17.47	14.24	4466.90
G58	IR68897A × RPHR619-2	E2A	104	84.75	15	22.92	3.24	177	90.11	17.55	19.07	6057.03
G58	IR68897A × RPHR619-2	E2B	97	90.09	13	19.91	2.71	158	87.79	16.12	17.03	5354.77
G58	IR68897A × RPHR619-2	E2C	105	86.36	13	20.60	2.88	171	87.91	16.22	12.92	4030.56
G59	IR68897A × RPHR1005	E1A	105	85.42	12	24.42	2.86	132	89.27	20.56	18.89	5993.22
G59	IR68897A × RPHR1005	E1B	97	85.21	11	23.45	2.52	130	87.84	20.85	17.96	5635.47
G59	IR68897A × RPHR1005	E1C	105	81.68	12	24.37	2.63	134	90.23	20.44	13.48	4217.47
G59	IR68897A × RPHR1005	E2A	102	83.38	13	25.06	2.82	142	88.93	20.52	18.32	5807.61
G59	IR68897A × RPHR1005	E2B	96	88.63	11	22.04	2.29	123	86.61	19.09	16.27	5105.35
G59	IR68897A × RPHR1005	E2C	104	84.92	10	22.74	2.46	136	86.73	19.19	12.17	3781.14
G60	IR68897A × RPHR517	E1A	101	87.91	11	22.61	3.24	229	90.08	16.99	19.35	6144.20
G60	IR68897A × RPHR517	E1B	94	87.91	11	21.64	2.90	226	88.65	17.28	18.42	5786.44

G60	IR68897A × RPHR517	E1C	102	84.12	11	22.56	3.01	231	91.04	16.87	13.95	4368.45
G60	IR68897A × RPHR517	E2A	99	86.01	12	23.25	3.21	239	89.74	16.95	18.78	5958.58
G60	IR68897A × RPHR517	E2B	92	91.33	10	20.23	2.67	219	87.42	15.52	16.73	5256.32
G60	IR68897A × RPHR517	E2C	101	87.38	10	20.93	2.84	233	87.54	15.62	12.63	3932.11
G61	IR68897A × IR40750R	E1A	113	83.07	12	23.06	2.77	124	90.54	19.89	19.37	6152.54
G61	IR68897A × IR40750R	E1B	106	83.04	11	22.09	2.43	122	89.11	20.18	18.44	5794.78
G61	IR68897A × IR40750R	E1C	114	79.25	12	23.01	2.54	127	91.50	19.77	13.97	4376.79
G61	IR68897A × IR40750R	E2A	111	81.17	13	23.70	2.74	135	90.20	19.85	18.80	5966.92
G61	IR68897A × IR40750R	E2B	104	86.46	11	20.68	2.20	115	87.88	18.42	16.75	5264.66
G61	IR68897A × IR40750R	E2C	113	82.49	10	21.38	2.37	128	88.00	18.52	12.65	3940.45
G62	IR68897A × 50-10	E1A	106	82.22	12	23.06	3.13	146	89.35	19.07	18.44	5845.64
G62	IR68897A × 50-10	E1B	99	82.07	11	22.09	2.78	144	87.92	19.36	17.51	5487.88
G62	IR68897A × 50-10	E1C	107	78.08	12	23.01	2.90	149	90.31	18.94	13.04	4069.89
G62	IR68897A × 50-10	E2A	103	80.32	13	23.70	3.09	156	89.01	19.03	17.87	5660.02
G62	IR68897A × 50-10	E2B	97	85.49	11	20.68	2.56	137	86.69	17.60	15.82	4957.76
G62	IR68897A × 50-10	E2C	106	81.32	11	21.38	2.73	150	86.82	17.70	11.72	3633.55
G63	IR68897A × C20R	E1A	103	93.91	13	23.30	3.01	163	90.44	19.76	26.85	8622.31
G63	IR68897A × C20R	E1B	96	93.96	12	22.33	2.67	161	89.01	20.05	25.93	8264.56
G63	IR68897A × C20R	E1C	104	90.08	13	23.25	2.78	166	91.40	19.63	21.45	6846.56
G63	IR68897A × C20R	E2A	101	92.01	14	23.94	2.98	174	90.10	19.72	26.29	8436.70
G63	IR68897A × C20R	E2B	94	97.38	12	20.93	2.44	154	87.77	18.28	24.24	7734.44
G63	IR68897A × C20R	E2C	103	93.62	12	21.62	2.61	167	87.90	18.39	20.13	6410.23
G64	IR68897A × RPHR695-1	E1A	105	84.79	13	22.22	2.54	144	85.83	16.57	18.39	5830.26
G64	IR68897A × RPHR695-1	E1B	98	85.04	12	21.25	2.19	141	84.40	16.86	17.47	5472.50
G64	IR68897A × RPHR695-1	E1C	106	80.98	12	22.17	2.31	146	86.79	16.45	12.99	4054.51
G64	IR68897A × RPHR695-1	E2A	103	82.75	13	22.86	2.50	154	85.49	16.53	17.82	5644.64
G64	IR68897A × RPHR695-1	E2B	96	88.46	11	19.84	1.96	134	83.17	15.10	15.78	4942.38
G64	IR68897A × RPHR695-1	E2C	104	84.22	11	20.54	2.14	148	83.29	15.20	11.67	3618.17
G65	IR68897A × IR-66R	E1A	108	93.18	12	24.55	3.82	238	90.01	18.91	35.22	11383.22
G65	IR68897A × IR-66R	E1B	101	93.02	11	23.58	3.48	236	88.58	19.20	34.29	11025.47

G65	IR68897A × IR-66R	E1C	109	89.23	12	24.50	3.59	241	90.97	18.79	29.82	9607.47
G65	IR68897A × IR-66R	E2A	105	91.28	13	25.19	3.78	248	89.67	18.87	34.65	11197.61
G65	IR68897A × IR-66R	E2B	99	96.44	11	22.17	3.25	229	87.35	17.44	32.60	10495.35
G65	IR68897A × IR-66R	E2C	108	92.50	10	22.87	3.42	242	87.47	17.54	28.50	9171.14
G66	IR68897A × Akshayadhan	E1A	105	88.49	11	23.20	3.31	120	84.67	19.35	25.16	8062.87
G66	IR68897A × Akshayadhan	E1B	97	88.08	10	22.23	2.97	118	83.24	19.64	24.23	7705.12
G66	IR68897A × Akshayadhan	E1C	105	84.41	11	23.15	3.08	123	85.63	19.23	19.76	6287.12
G66	IR68897A × Akshayadhan	E2A	102	86.45	12	23.84	3.27	131	84.33	19.31	24.59	7877.26
G66	IR68897A × Akshayadhan	E2B	96	91.50	10	20.82	2.74	111	82.01	17.88	22.54	7175.00
G66	IR68897A × Akshayadhan	E2C	104	87.65	9	21.52	2.91	124	82.14	17.98	18.44	5850.79
G67	IR68897A × SG27-105	E1A	102	94.66	12	23.11	2.78	118	73.00	18.74	21.14	6737.83
G67	IR68897A × SG27-105	E1B	95	94.74	11	22.14	2.44	116	71.57	19.03	20.22	6380.07
G67	IR68897A × SG27-105	E1C	102	90.87	12	23.06	2.55	121	73.96	18.62	15.74	4962.08
G67	IR68897A × SG27-105	E2A	99	92.76	12	23.75	2.75	129	72.66	18.70	20.57	6552.22
G67	IR68897A × SG27-105	E2B	93	98.16	11	20.73	2.21	109	70.34	17.27	18.53	5849.96
G67	IR68897A × SG27-105	E2C	101	94.13	10	21.42	2.38	122	70.46	17.37	14.42	4525.74
G68	IR68897A × 363-5	E1A	107	85.22	11	22.80	3.33	159	83.48	18.87	24.58	7870.83
G68	IR68897A × 363-5	E1B	100	85.47	11	21.83	2.99	157	82.05	19.16	23.65	7513.07
G68	IR68897A × 363-5	E1C	107	81.11	11	22.75	3.10	162	84.44	18.75	19.18	6095.08
G68	IR68897A × 363-5	E2A	104	83.18	12	23.44	3.30	170	83.14	18.83	24.01	7685.22
G68	IR68897A × 363-5	E2B	98	88.89	10	20.42	2.76	150	80.82	17.40	21.96	6982.96
G68	IR68897A × 363-5	E2C	106	84.35	10	21.12	2.93	163	80.94	17.50	17.86	5658.74
G69	IR68897A × RPHR611-1	E1A	106	89.49	12	22.22	3.43	196	82.46	14.88	19.25	6112.11
G69	IR68897A × RPHR611-1	E1B	99	89.23	11	21.25	3.09	193	81.03	15.17	18.32	5754.36
G69	IR68897A × RPHR611-1	E1C	107	85.54	12	22.17	3.20	198	83.42	14.76	13.85	4336.36
G69	IR68897A × RPHR611-1	E2A	104	87.60	13	22.86	3.39	206	82.12	14.84	18.68	5926.50
G69	IR68897A × RPHR611-1	E2B	97	92.65	11	19.85	2.86	187	79.80	13.41	16.63	5224.24
G69	IR68897A × RPHR611-1	E2C	106	88.80	11	20.54	3.03	200	79.92	13.51	12.53	3900.03
G70	IR68897A × IBL57	E1A	103	84.01	11	23.32	3.11	175	87.22	19.03	28.03	9011.26
G70	IR68897A × IBL57	E1B	97	84.26	10	22.35	2.76	173	85.79	19.32	27.11	8653.50

G70	IR68897A × IBL57	E1C	104	80.10	11	23.27	2.87	178	88.18	18.90	22.63	7235.50
G70	IR68897A × IBL57	E2A	101	82.11	12	23.96	3.07	186	86.88	18.99	27.46	8825.64
G70	IR68897A × IBL57	E2B	95	87.68	10	20.95	2.53	166	84.56	17.56	25.42	8123.38
G70	IR68897A × IBL57	E2C	103	83.34	10	21.64	2.70	179	84.68	17.66	21.31	6799.17
G71	IR68897A × BK-49-180	E1A	97	79.97	11	22.74	2.75	146	90.32	19.81	19.77	6284.17
G71	IR68897A × BK-49-180	E1B	90	79.93	11	21.77	2.41	144	88.89	20.10	18.84	5926.42
G71	IR68897A × BK-49-180	E1C	97	76.18	11	22.69	2.52	149	91.28	19.69	14.37	4508.42
G71	IR68897A × BK-49-180	E2A	94	78.21	12	23.38	2.72	157	89.98	19.77	19.20	6098.56
G71	IR68897A × BK-49-180	E2B	88	83.35	10	20.37	2.18	137	87.66	18.34	17.15	5396.30
G71	IR68897A × BK-49-180	E2C	96	79.42	10	21.06	2.35	151	87.78	18.44	13.05	4072.09
G72	IR68897A × RPHR1096	E1A	115	96.73	11	24.14	3.92	173	87.12	20.81	29.22	9403.68
G72	IR68897A × RPHR1096	E1B	108	96.69	11	23.17	3.58	171	85.69	21.10	28.30	9045.92
G72	IR68897A × RPHR1096	E1C	116	93.03	11	24.09	3.69	175	88.08	20.69	23.82	7627.93
G72	IR68897A × RPHR1096	E2A	113	94.69	12	24.78	3.89	183	86.78	20.77	28.65	9218.07
G72	IR68897A × RPHR1096	E2B	106	100.11	10	21.76	3.35	164	84.46	19.34	26.61	8515.81
G72	IR68897A × RPHR1096	E2C	115	96.31	10	22.46	3.52	177	84.59	19.44	22.50	7191.59
G73	APMS6B	E1A	112	97.81	13	23.18	3.89	154	88.78	18.12	22.00	7020.53
G73	APMS6B	E1B	105	97.82	12	22.21	3.55	152	87.35	18.41	21.08	6662.77
G73	APMS6B	E1C	113	94.29	13	23.13	3.66	157	89.74	18.00	16.60	5244.78
G73	APMS6B	E2A	110	95.77	14	23.82	3.85	165	88.44	18.08	21.43	6834.92
G73	APMS6B	E2B	103	101.26	12	20.80	3.32	145	86.11	16.65	19.39	6132.66
G73	APMS6B	E2C	111	97.40	12	21.50	3.49	158	86.24	16.75	15.28	4808.44
G74	IR58025B	E1A	116	106.39	11	21.97	2.96	155	89.13	19.31	26.01	8344.01
G74	IR58025B	E1B	109	106.42	10	21.00	2.62	152	87.70	19.60	25.08	7986.26
G74	IR58025B	E1C	117	102.49	11	21.92	2.73	157	90.09	19.19	20.61	6568.26
G74	IR58025B	E2A	114	104.35	12	22.61	2.92	165	88.79	19.27	25.44	8158.40
G74	IR58025B	E2B	107	109.88	10	19.59	2.39	146	86.47	17.84	23.40	7456.14
G74	IR58025B	E2C	116	105.60	10	20.29	2.56	159	86.59	17.94	19.29	6131.93
G75	IR79156B	E1A	118	105.58	9	21.86	2.77	133	86.09	19.04	23.07	7374.82
G75	IR79156B	E1B	111	105.23	8	20.89	2.43	130	84.66	19.33	22.15	7017.07

G75	IR79156B	E1C	118	101.61	9	21.82	2.54	135	87.05	18.92	17.67	5599.07
G75	IR79156B	E2A	115	103.68	10	22.50	2.73	143	85.75	19.00	22.50	7189.21
G75	IR79156B	E2B	109	108.65	8	19.49	2.20	124	83.43	17.57	20.46	6486.95
G75	IR79156B	E2C	117	104.72	7	20.18	2.37	137	83.56	17.67	16.35	5162.74
G76	IR68897B	E1A	111	98.67	9	22.64	3.40	146	87.65	22.32	22.98	7344.21
G76	IR68897B	E1B	104	98.63	9	21.67	3.06	144	86.23	22.61	22.06	6986.45
G76	IR68897B	E1C	111	94.82	9	22.59	3.17	149	88.62	22.19	17.58	5568.45
G76	IR68897B	E2A	108	96.63	10	23.28	3.36	157	87.32	22.28	22.41	7158.59
G76	IR68897B	E2B	102	102.06	8	20.27	2.83	137	84.99	20.84	20.37	6456.33
G76	IR68897B	E2C	110	98.10	8	20.96	3.00	150	85.12	20.95	16.26	5132.12
G77	BCW-56	E1A	111	94.48	11	21.11	2.68	119	87.57	17.65	21.21	6759.83
G77	BCW-56	E1B	104	94.76	11	20.14	2.34	117	86.14	17.94	20.28	6402.07
G77	BCW-56	E1C	111	91.05	11	21.06	2.45	122	88.53	17.53	15.81	4984.08
G77	BCW-56	E2A	108	92.44	12	21.75	2.64	130	87.23	17.61	20.64	6574.22
G77	BCW-56	E2B	102	98.18	10	18.73	2.11	110	84.91	16.18	18.60	5871.96
G77	BCW-56	E2C	110	94.33	10	19.43	2.28	123	85.03	16.28	14.49	4547.74
G78	EPLT104	E1A	110	99.76	12	24.64	2.58	99	87.95	21.24	28.73	8632.96
G78	EPLT104	E1B	103	99.94	11	23.67	2.24	97	86.53	21.53	27.81	8275.21
G78	EPLT104	E1C	110	95.96	11	24.59	2.35	102	88.91	21.12	23.33	6857.21
G78	EPLT104	E2A	107	97.72	12	25.28	2.54	109	87.62	21.20	28.17	8447.35
G78	EPLT104	E2B	101	103.38	11	22.26	2.01	90	85.29	19.77	26.12	7745.09
G78	EPLT104	E2C	109	99.24	10	22.96	2.18	103	85.42	19.87	22.02	6420.88
G79	KMR3	E1A	116	98.99	11	23.66	2.75	139	89.52	18.08	24.77	7935.46
G79	KMR3	E1B	109	98.79	11	22.69	2.40	137	88.09	18.37	23.85	7577.70
G79	KMR3	E1C	117	94.84	11	23.61	2.51	142	90.48	17.96	19.37	6159.70
G79	KMR3	E2A	114	96.95	12	24.30	2.71	150	89.18	18.04	24.20	7749.84
G79	KMR3	E2B	107	102.21	10	21.29	2.17	130	86.86	16.61	22.16	7047.58
G79	KMR3	E2C	115	98.12	10	21.98	2.34	143	86.98	16.71	18.05	5723.37
G80	RPHR 619-2	E1A	113	86.94	11	23.47	3.37	112	90.45	17.40	23.52	7522.68
G80	RPHR 619-2	E1B	106	86.98	11	22.50	3.03	110	89.02	17.69	22.59	7164.92

G80	RPHR 619-2	E1C	113	83.06	11	23.42	3.14	114	91.41	17.27	18.12	5746.93
G80	RPHR 619-2	E2A	110	84.90	12	24.11	3.34	122	90.11	17.36	22.95	7337.07
G80	RPHR 619-2	E2B	104	90.40	10	21.09	2.80	103	87.79	15.93	20.91	6634.81
G80	RPHR 619-2	E2C	112	86.30	10	21.79	2.97	116	87.91	16.03	16.80	5310.59
G81	RPHR1005	E1A	111	85.83	10	22.73	3.70	187	83.67	16.84	19.66	6247.32
G81	RPHR1005	E1B	104	85.55	10	21.76	3.36	184	82.24	17.13	18.73	5889.57
G81	RPHR1005	E1C	112	82.33	10	22.68	3.47	189	84.63	16.72	14.26	4471.57
G81	RPHR1005	E2A	109	83.79	11	23.37	3.67	197	83.33	16.80	19.09	6061.71
G81	RPHR1005	E2B	103	88.97	9	20.35	3.13	178	81.00	15.37	17.04	5359.45
G81	RPHR1005	E2C	110	85.60	9	21.05	3.30	191	81.13	15.47	12.94	4035.24
G82	RPHR517	E1A	110	101.62	11	23.05	2.96	135	85.53	18.42	24.85	7961.40
G82	RPHR517	E1B	103	101.66	11	22.08	2.62	133	84.10	18.71	23.92	7603.64
G82	RPHR517	E1C	111	97.72	11	23.00	2.73	138	86.49	18.30	19.45	6185.65
G82	RPHR517	E2A	108	99.58	12	23.69	2.92	145	85.19	18.38	24.28	7775.78
G82	RPHR517	E2B	101	105.10	10	20.68	2.39	126	82.87	16.95	22.24	7073.52
G82	RPHR517	E2C	110	101.01	10	21.37	2.56	139	82.99	17.05	18.13	5749.31
G83	IR40750R	E1A	121	99.19	10	22.08	2.94	182	86.14	17.39	17.43	5511.42
G83	IR40750R	E1B	113	99.16	9	21.11	2.60	180	84.71	17.68	16.50	5153.67
G83	IR40750R	E1C	121	95.37	10	22.04	2.71	185	87.10	17.26	12.03	3735.67
G83	IR40750R	E2A	118	97.44	11	22.72	2.90	193	85.80	17.35	16.86	5325.81
G83	IR40750R	E2B	112	102.58	9	19.71	2.37	173	83.48	15.92	14.81	4623.55
G83	IR40750R	E2C	120	98.65	8	20.40	2.54	186	83.60	16.02	10.71	3299.34
G84	50-10	E1A	112	102.86	12	22.80	3.05	165	82.04	19.94	23.03	7362.52
G84	50-10	E1B	104	102.77	12	21.83	2.71	163	80.62	20.23	22.11	7004.76
G84	50-10	E1C	112	98.87	12	22.75	2.82	168	83.00	19.82	17.63	5586.77
G84	50-10	E2A	109	100.82	13	23.44	3.01	176	81.71	19.90	22.47	7176.91
G84	50-10	E2B	102	106.21	11	20.42	2.48	156	79.38	18.47	20.42	6474.65
G84	50-10	E2C	110	102.15	10	21.12	2.65	170	79.51	18.57	16.32	5150.43
G85	C20R	E1A	119	99.92	11	23.56	3.22	145	86.48	19.24	19.34	6141.91
G85	C20R	E1B	112	100.00	11	22.59	2.87	143	85.05	19.53	18.41	5784.15

G85	C20R	E1C	119	96.19	11	23.51	2.99	148	87.44	19.12	13.94	4366.15
G85	C20R	E2A	116	97.88	12	24.20	3.18	155	86.14	19.20	18.77	5956.29
G85	C20R	E2B	110	103.42	10	21.18	2.64	136	83.82	17.77	16.72	5254.03
G85	C20R	E2C	118	99.48	10	21.88	2.82	149	83.94	17.87	12.62	3929.82
G86	RPHR 695-1	E1A	115	104.52	10	22.98	2.96	145	88.36	18.04	21.76	6940.96
G86	RPHR 695-1	E1B	108	104.56	10	22.01	2.61	143	86.93	18.33	20.83	6583.21
G86	RPHR 695-1	E1C	116	100.59	10	22.93	2.73	148	89.32	17.92	16.36	5165.21
G86	RPHR 695-1	E2A	113	102.48	11	23.62	2.92	155	88.02	18.00	21.19	6755.35
G86	RPHR 695-1	E2B	106	108.00	9	20.60	2.38	136	85.70	16.57	19.14	6053.09
G86	RPHR 695-1	E2C	115	103.87	9	21.30	2.56	149	85.82	16.67	15.04	4728.88
G87	IR-66R	E1A	112	96.22	12	22.45	2.79	146	88.95	18.14	23.53	7524.97
G87	IR-66R	E1B	105	96.46	12	21.48	2.45	143	87.52	18.43	22.60	7167.22
G87	IR-66R	E1C	113	92.48	12	22.40	2.56	148	89.91	18.02	18.13	5749.22
G87	IR-66R	E2A	110	94.18	13	23.09	2.75	156	88.61	18.10	22.96	7339.36
G87	IR-66R	E2B	103	99.90	11	20.07	2.22	137	86.29	16.67	20.91	6637.10
G87	IR-66R	E2C	112	95.76	11	20.77	2.39	150	86.41	16.77	16.81	5312.89
G88	Akshayadhan	E1A	116	104.41	11	22.75	3.42	166	88.50	17.04	28.69	9227.31
G88	Akshayadhan	E1B	108	104.37	11	21.78	3.08	164	87.07	17.33	27.76	8869.56
G88	Akshayadhan	E1C	116	100.80	11	22.71	3.19	169	89.46	16.92	23.29	7451.56
G88	Akshayadhan	E2A	113	102.37	12	23.39	3.39	177	88.16	17.00	28.12	9041.70
G88	Akshayadhan	E2B	106	107.81	10	20.38	2.85	157	85.84	15.57	26.07	8339.44
G88	Akshayadhan	E2C	115	103.91	9	21.07	3.02	170	85.96	15.67	21.97	7015.23
G89	SG-27-105	E1A	115	97.32	13	22.31	3.04	176	89.17	20.62	19.77	6285.46
G89	SG-27-105	E1B	108	96.98	12	21.34	2.69	174	87.74	20.91	18.85	5927.70
G89	SG-27-105	E1C	116	93.49	12	22.26	2.81	179	90.13	20.50	14.37	4509.70
G89	SG-27-105	E2A	113	95.28	14	22.95	3.00	186	88.83	20.58	19.20	6099.84
G89	SG-27-105	E2B	106	100.40	12	19.94	2.47	167	86.51	19.15	17.16	5397.58
G89	SG-27-105	E2C	114	96.77	11	20.63	2.64	180	86.63	19.25	13.05	4073.37
G90	363-5	E1A	107	88.80	12	21.63	3.22	151	89.61	16.69	20.93	6667.43
G90	363-5	E1B	100	88.51	12	20.66	2.88	149	88.18	16.98	20.00	6309.67

G90	363-5	E1C	107	84.71	12	21.59	2.99	154	90.57	16.57	15.53	4891.68
G90	363-5	E2A	104	86.76	13	22.27	3.18	161	89.27	16.65	20.36	6481.82
G90	363-5	E2B	98	91.93	11	19.26	2.65	142	86.95	15.22	18.31	5779.56
G90	363-5	E2C	106	87.84	11	19.95	2.82	155	87.07	15.32	14.21	4455.34
G91	RPHR 611-1	E1A	115	105.47	10	23.21	3.35	162	84.84	18.80	26.04	8353.64
G91	RPHR 611-1	E1B	108	105.44	10	22.24	3.01	159	83.42	19.09	25.12	7995.88
G91	RPHR 611-1	E1C	115	101.82	10	23.16	3.12	164	85.80	18.68	20.64	6577.89
G91	RPHR 611-1	E2A	112	103.43	11	23.85	3.31	172	84.51	18.76	25.47	8168.02
G91	RPHR 611-1	E2B	106	108.90	9	20.84	2.78	153	82.18	17.33	23.43	7465.76
G91	RPHR 611-1	E2C	114	104.75	8	21.53	2.95	166	82.31	17.43	19.32	6141.55
G92	IBL57	E1A	109	98.09	13	22.70	3.32	150	89.39	16.74	27.07	8694.18
G92	IBL57	E1B	102	97.98	13	21.73	2.97	148	87.96	17.03	26.15	8336.42
G92	IBL57	E1C	109	94.48	13	22.66	3.09	153	90.35	16.62	21.67	6918.43
G92	IBL57	E2A	106	96.04	14	23.34	3.28	160	89.05	16.70	26.50	8508.57
G92	IBL57	E2B	100	101.40	12	20.33	2.74	141	86.73	15.27	24.46	7806.31
G92	IBL57	E2C	108	97.59	12	21.02	2.92	154	86.85	15.37	20.35	6482.09
G93	BK49-180	E1A	80	86.08	9	21.18	1.73	137	82.01	16.40	18.97	6021.27
G93	BK49-180	E1B	73	86.06	9	20.21	1.39	135	80.58	16.69	18.05	5663.52
G93	BK49-180	E1C	81	82.80	9	21.13	1.50	140	82.97	16.28	13.57	4245.52
G93	BK49-180	E2A	78	84.33	10	21.82	1.69	148	81.67	16.36	18.40	5835.66
G93	BK49-180	E2B	71	89.48	9	18.81	1.16	128	79.34	14.93	16.36	5133.40
G93	BK49-180	E2C	80	86.00	8	19.50	1.33	141	79.47	15.03	12.25	3809.19
G94	RPHR1096	E1A	105	103.29	12	23.48	2.94	199	88.31	18.75	22.80	7283.80
G94	RPHR1096	E1B	98	103.11	11	22.51	2.59	197	86.88	19.04	21.87	6926.04
G94	RPHR1096	E1C	106	99.09	12	23.43	2.71	202	89.27	18.63	17.40	5508.05
G94	RPHR1096	E2A	103	101.25	13	24.12	2.90	210	87.97	18.71	22.23	7098.18
G94	RPHR1096	E2B	96	106.55	11	21.10	2.37	190	85.64	17.28	20.18	6395.92
G94	RPHR1096	E2C	104	102.70	10	21.79	2.54	204	85.77	17.38	16.08	5071.71
G95	PA6201	E1A	106	89.74	13	23.86	2.90	186	87.29	18.78	22.53	7194.33
G95	PA6201	E1B	99	89.60	12	22.89	2.55	184	85.86	19.07	21.60	6836.57

G95	PA6201	E1C	107	85.87	12	23.81	2.67	189	88.25	18.65	17.13	5418.58
G95	PA6201	E2A	104	87.70	13	24.50	2.86	196	86.95	18.74	21.96	7008.72
G95	PA6201	E2B	97	93.02	11	21.48	2.32	177	84.63	17.30	19.91	6306.46
G95	PA6201	E2C	105	89.14	11	22.18	2.50	190	84.75	17.41	15.81	4982.24
G96	NDR359	E1A	106	92.33	13	23.97	3.74	152	88.75	19.06	22.69	7163.35
G96	NDR359	E1B	99	92.22	13	23.00	3.40	149	87.32	19.35	21.76	6805.59
G96	NDR359	E1C	106	88.54	13	23.92	3.51	154	89.71	18.93	17.28	5387.60
G96	NDR359	E2A	103	90.43	14	24.61	3.70	162	88.41	19.02	22.12	6977.73
G96	NDR359	E2B	97	95.65	12	21.59	3.17	143	86.09	17.59	20.07	6275.47
G96	NDR359	E2C	105	91.80	12	22.29	3.34	156	86.21	17.69	15.97	4951.26
G97	BPT5204	E1A	118	103.39	10	22.86	2.90	121	88.48	20.01	22.71	7253.09
G97	BPT5204	E1B	111	103.43	10	21.89	2.56	118	87.06	20.30	21.78	6895.33
G97	BPT5204	E1C	118	99.76	10	22.82	2.67	123	89.45	19.89	17.30	5477.34
G97	BPT5204	E2A	115	101.35	11	23.50	2.87	131	88.15	19.97	22.14	7067.47
G97	BPT5204	E2B	109	106.87	9	20.49	2.33	112	85.82	18.54	20.09	6365.21
G97	BPT5204	E2C	117	102.87	9	21.18	2.50	125	85.95	18.64	15.99	5041.00
G98	KRH2	E1A	113	90.71	12	21.94	3.27	112	81.70	19.41	23.33	7460.81
G98	KRH2	E1B	106	90.90	11	20.97	2.93	110	80.27	19.70	22.41	7103.05
G98	KRH2	E1C	114	87.11	11	21.90	3.04	115	82.66	19.29	17.93	5685.05
G98	KRH2	E2A	111	88.67	12	22.58	3.23	123	81.36	19.37	22.77	7275.19
G98	KRH2	E2B	104	94.32	11	19.57	2.70	103	79.04	17.94	20.72	6572.93
G98	KRH2	E2C	112	90.37	10	20.26	2.87	117	79.16	18.04	16.61	5248.72
G99	DRRH3	E1A	113	101.72	11	22.87	3.20	141	88.09	17.33	24.79	7941.14
G99	DRRH3	E1B	106	101.67	10	21.90	2.86	139	86.67	17.62	23.86	7583.38
G99	DRRH3	E1C	113	98.15	10	22.82	2.97	144	89.06	17.21	19.39	6165.39
G99	DRRH3	E2A	110	99.67	11	23.51	3.16	152	87.76	17.29	24.22	7755.52
G99	DRRH3	E2B	104	105.09	9	20.49	2.63	132	85.43	15.86	22.17	7053.26
G99	DRRH3	E2C	112	101.26	9	21.18	2.80	145	85.56	15.96	18.07	5729.05
G100	IR64	E1A	113	94.76	11	22.55	2.25	106	87.74	20.73	17.32	5475.76
G100	IR64	E1B	106	94.44	10	21.58	1.91	104	86.31	21.02	16.39	5118.01

G100	IR64	E1C	113	91.34	11	22.50	2.02	109	88.70	20.61	11.92	3700.01
G100	IR64	E2A	110	92.72	12	23.19	2.22	117	87.40	20.69	16.75	5290.15
G100	IR64	E2B	104	97.86	10	20.18	1.68	97	85.08	19.26	14.70	4587.89
G100	IR64	E2C	112	94.45	9	20.87	1.85	110	85.21	19.36	10.60	3263.68
G101	PA6444	E1A	109	105.64	10	24.19	3.08	150	84.96	17.70	22.83	7294.43
G101	PA6444	E1B	102	105.58	9	23.22	2.74	148	83.54	17.99	21.90	6936.67
G101	PA6444	E1C	109	102.28	10	24.14	2.85	153	85.92	17.58	17.43	5518.68
G101	PA6444	E2A	106	103.60	11	24.83	3.05	161	84.63	17.66	22.26	7108.82
G101	PA6444	E2B	100	109.02	9	21.82	2.51	141	82.30	16.23	20.21	6406.56
G101	PA6444	E2C	107	105.22	8	22.51	2.68	154	82.43	16.33	16.11	5082.34
G102	Nagina22 (N22)	E1A	117	91.87	13	21.41	2.40	131	82.02	18.92	23.42	7487.76
G102	Nagina22 (N22)	E1B	110	92.12	12	20.44	2.05	129	80.59	19.21	22.49	7130.00
G102	Nagina22 (N22)	E1C	118	88.28	12	21.36	2.17	134	82.98	18.80	18.01	5712.00
G102	Nagina22 (N22)	E2A	115	89.97	14	22.05	2.36	141	81.68	18.88	22.85	7302.14
G102	Nagina22 (N22)	E2B	108	95.54	12	19.04	1.82	122	79.36	17.45	20.80	6599.88
G102	Nagina22 (N22)	E2C	116	91.54	11	19.73	2.00	135	79.48	17.55	16.70	5275.67
G103	Azucena	E1A	111	90.52	9	21.98	2.46	124	82.23	17.59	20.93	6666.42
G103	Azucena	E1B	104	90.60	9	21.01	2.11	122	80.80	17.89	20.00	6308.67
G103	Azucena	E1C	111	86.93	9	21.94	2.23	127	83.19	17.47	15.52	4890.67
G103	Azucena	E2A	109	88.48	10	22.62	2.42	134	81.89	17.55	20.36	6480.81
G103	Azucena	E2B	102	94.02	8	19.61	1.88	115	79.57	16.12	18.31	5778.55
G103	Azucena	E2C	110	90.19	8	20.30	2.06	128	79.69	16.22	14.21	4454.34

Supplementary Table 3. List of genotypes used in this study.

CODE	GENOTYPE	CODE	GENOTYPE	CODE	GENOTYPE	CODE	GENOTYPE
G1	APMS6A × BCW56	G27	IR58025A × C20R	G53	IR79156A × BK-49-180	G79	KMR3
G2	APMS6A × EPLT104	G28	IR58025A × RPHR695-1	G54	IR79156A × RPHR1096	G80	RPHR 619-2
G3	APMS6A × KMR3	G29	IR58025A × IR-66R	G55	IR68897A × BCW56	G81	RPHR1005
G4	APMS6A × RPHR619-2	G30	IR58025A × Akshayadhan	G56	IR68897A × EPLT104	G82	RPHR517
G5	APMS6A × RPHR1005	G31	IR58025A × SG27-105	G57	IR68897A × KMR3	G83	IR40750R
G6	APMS6A × RPHR517	G32	IR58025A × 363-5	G58	IR68897A × RPHR619-2	G84	50-10
G7	APMS6A × IR40750R	G33	IR58025A × RPHR611-1	G59	IR68897A × RPHR1005	G85	C20R
G8	APMS6A × 50-10	G34	IR58025A × IBL57	G60	IR68897A × RPHR517	G86	RPHR 695-1
G9	APMS6A × C20R	G35	IR58025A × BK-49-180	G61	IR68897A × IR40750R	G87	IR-66R
G10	APMS6A × RPHR695-1	G36	IR58025A × RPHR1096	G62	IR68897A × 50-10	G88	Akshayadhan
G11	APMS6A × IR-66R	G37	IR79156A × BCW56	G63	IR68897A × C20R	G89	SG-27-105
G12	APMS6A × Akshayadhan	G38	IR79156A × EPLT104	G64	IR68897A × RPHR695-1	G90	363-5
G13	APMS6A × SG27-105	G39	IR79156A × KMR3	G65	IR68897A × IR-66R	G91	RPHR 611-1
G14	APMS6A × 363-5	G40	IR79156A × RPHR619-2	G66	IR68897A × Akshayadhan	G92	IBL57
G15	APMS6A × RPHR611-1	G41	IR79156A × RPHR1005	G67	IR68897A × SG27-105	G93	BK49-180
G16	APMS6A × IBL57	G42	IR79156A × RPHR517	G68	IR68897A × 363-5	G94	RPHR1096
G17	APMS6A × BK-49-180	G43	IR79156A × IR40750R	G69	IR68897A × RPHR611-1	G95	PA6201
G18	APMS6A × RPHR1096	G44	IR79156A × 50-10	G70	IR68897A × IBL57	G96	NDR359
G19	IR58025A × BCW56	G45	IR79156A × C20R	G71	IR68897A × BK-49-180	G97	BPT5204
G20	IR58025A × EPLT104	G46	IR79156A × RPHR695-1	G72	IR68897A × RPHR1096	G98	KRH2
G21	IR58025A × KMR3	G47	IR79156A × IR-66R	G73	APMS6B	G99	DRRH3
G22	IR58025A × RPHR619-2	G48	IR79156A × Akshayadhan	G74	IR58025B	G100	IR64
G23	IR58025A × RPHR1005	G49	IR79156A × SG27-105	G75	IR79156B	G101	PA6444
G24	IR58025A × RPHR517	G50	IR79156A × 363-5	G76	IR68897B	G102	Nagina22 (N22)
G25	IR58025A × IR40750R	G51	IR79156A × RPHR611-1	G77	BCW-56	G103	Azucena

G26	IR58025A × 50-10	G52	IR79156A × IBL57	G78	EPLT104

Supplementary Table 4. ANOVA and Mean Sum of Squares on G, E and G × E for all the studied traits.

**Source of Variation**	**df**	**DFF (Days)**	**PH (cm)**	**NRT**	**PL (cm)**	**PW (grams)**	**GP**	**SF (%)**	**TW (grams)**	**SPY (grams)**	**YIELD (kg)**
**Environments (E)**	5	3295.4280	1203.0066	175.6086	285.0424	10.8925	11608.2733	488.1312	131.1832	1693.7106	179622986.3
**Replication (E)**	6	49.9118	3.9405	12.3628	7.8278	0.4405	1537.5643	11.7753	2.2541	56.4651	5467674.541
**Genotypes (G)**	102	428.2908	692.3235	15.9982	9.9684	1.7336	7337.7294	124.1726	25.1293	138.5199	14040074.34
**G × E**	510	24.2316	25.1198	5.2738	2.5375	0.3568	550.4086	22.2462	3.1756	14.6437	1506389.389
**Pooled Error**	612	23.1798	24.2132	4.9997	3.8767	0.5655	1577.5135	30.7016	7.8937	20.8993	2192372.739
**Total**	1235										
**Chi square Value (Bartlett's Test)**	102	376.82	479.64	416.98	423.01	429.47	683.92	846.51	566.42	548.47	543.07
**W Value Test for Normality (Shapiro-Wilk)**		0.9945	0.9959	0.9910	0.9940	0.9919	0.9937	0.9437	0.9905	0.9953	0.9957
